# Nicotinamide Adenine Dinucleotide Supplementation to Alleviate Heart Failure: A Mitochondrial Dysfunction Perspective

**DOI:** 10.3390/nu17111855

**Published:** 2025-05-29

**Authors:** Fan Yu, Huiying Zhao, Lu Luo, Wei Wu

**Affiliations:** 1 School of Exercise and Health, Shanghai University of Sport, Shanghai 200438, China; 13734934551@163.com (F.Y.); zhaohy1237@163.com (H.Z.); 2 Department of Anesthesiology, EYE & ENT Hospital of Fudan University, Shanghai 200032, China; 3 School of Athletic Performance, Shanghai University of Sport, Shanghai 200438, China

**Keywords:** heart failure, nicotinamide adenine dinucleotide, mitochondrial dysfunction, redox, ATP

## Abstract

Heart failure represents the terminal stage in the development of many cardiovascular diseases, and its pathological mechanisms are closely related to disturbances in energy metabolism and mitochondrial dysfunction in cardiomyocytes. In recent years, nicotinamide adenine dinucleotide (NAD^+^), a core coenzyme involved in cellular energy metabolism and redox homeostasis, has been shown to potentially ameliorate heart failure through the regulation of mitochondrial function. This review systematically investigates four core mechanisms of mitochondrial dysfunction in heart failure: imbalance of mitochondrial dynamics, excessive accumulation of reactive oxygen species (ROS) leading to oxidative stress injury, dysfunction of mitochondrial autophagy, and disturbance of Ca^2+^ homeostasis. These abnormalities collectively exacerbate the progression of heart failure by disrupting ATP production and inducing apoptosis and myocardial fibrosis. NAD^+^ has been shown to regulate mitochondrial biosynthesis and antioxidant defences through the activation of the deacetylase family (e.g., silent information regulator 2 homolog 1 (SIRT1) and SIRT3) and to increase mitochondrial autophagy to remove damaged mitochondria, thus restoring energy metabolism and redox balance in cardiomyocytes. In addition, the inhibition of NAD^+^-degrading enzymes (e.g., poly ADP-ribose polymerase (PARP), cluster of differentiation 38 (CD38), and selective androgen receptor modulators (SARMs)) increases the tissue intracellular NAD^+^ content, and supplementation with NAD^+^ precursors (e.g., β-nicotinamide mononucleotide (NMN), nicotinamide riboside, etc.) also significantly elevates myocardial NAD^+^ levels to ameliorate heart failure. This study provides a theoretical basis for understanding the central role of NAD^+^ in mitochondrial homeostasis and for the development of targeted therapies for heart failure.

## 1. Introduction

Heart failure is a multifactorial progressive disease, typically defined as ‘the inability of the heart to pump enough blood to meet the body’s needs’ or ‘structural/functional ventricular anomalies resulting in a decrease in cardiac output or an increase in intracardiac pressure at rest or during stress’ [1]. Heart failure represents the end stage of several cardiovascular diseases, including arrhythmias, myocardial infarction, myocarditis, and hypertension [2], the prevalence of which is associated mainly with an ageing population, diabetes mellitus, obesity, and ischemic heart disease [3,4]. It is estimated that in 2019, approximately 60 million people worldwide suffered from heart failure, representing a nearly 55% increase from 1990. The global prevalence of heart failure in adults is 1–3%, making it the fastest-growing cardiovascular disease in the world [5,6,7]. This imposes a serious economic burden on society. In the treatment guidelines for heart failure, the following pharmaceutical combinations are recommended: β-adrenergic receptor blockers, angiotensin receptor-neprilysin inhibitors [8], mineralocorticoid receptor antagonists (MRA), and sodium-dependent glucose transporter 2 (SGLT2) inhibitors [9]. However, these pharmaceuticals have been reported to induce a range of adverse effects that have the potential to compromise their clinical efficacy. For example, β-adrenergic receptor blockers have been reported to induce arrhythmias, ARNI have been associated with hypotension and renal insufficiency, and MRA have been linked to hyperkalaemia [10]. Despite significant advances in the treatment of heart failure, the overall prognosis for patients remains poor, and substantial halting of disease progression has yet to be achieved [11]. Consequently, researchers must identify new therapeutic targets and strategies for the study of heart failure.

**Figure 1:**
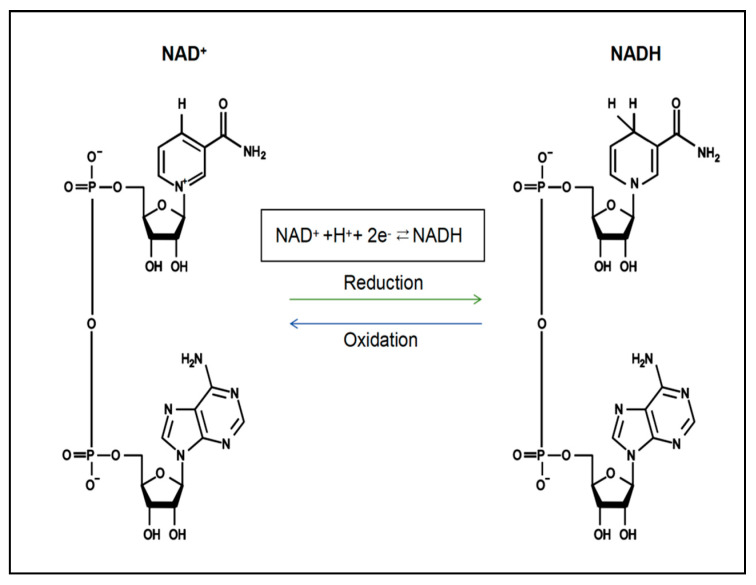
The chemical structure and conversion of NAD^+^ and NADH.

Nicotinamide adenine dinucleotide (NAD^+^) is an important coenzyme and energy metabolism centre for intracellular redox reactions that directly or indirectly affect many key physiological functions in the cell, including cellular metabolism, DNA damage, redox reactions, mitochondrial function, inflammation and cellular ageing [12,13]. NAD^+^ is abundant in the human body and is continuously synthesised, degraded, and recycled in major organelles, such as the nucleus, cytoplasm, Golgi apparatus, and peroxisome, to maintain stable intracellular NAD^+^ levels and physiological metabolic functions [14,15]. NAD^+^ levels are maintained primarily by three separate biosynthetic pathways: the de novo synthesis pathway (tryptophan), the Preiss–Handler pathway (nicotinic acid (NA)) and the salvage pathway (nicotinamide (NAM), nicotinamide mononucleotide (NMN), etc.) [16]. The reformed NAD^+^ in these synthetic pathways accepts hydride to produce the reduced form of nicotinamide adenine dinucleotide (NADH) (Figure 1), which drives various metabolic processes, including the tricarboxylic acid cycle (TCA cycle), oxidative phosphorylation and cellular glycolysis, to mediate energy metabolism [16]. In contrast, the NAD^+^ depletion pathway is an irreversible biodegradation process [17], which ultimately causes an imbalance in the homeostasis of multiple intracellular metabolic pathways, leading to the development of disease [18]. The levels of NAD^+^ in various cell tissues decrease with age, resulting in irreversible cell cycle arrest, which induces the onset of cellular senescence and further reduces the levels of NAD^+^ [19]. Several studies have shown that NAD^+^ depletion is an important cause of heart failure [20]. Increasing NAD^+^ levels has been suggested as a good strategy for the treatment of heart failure [21], but the exact mechanism of action is still unclear.

The heart is a highly energy-consuming organ, with the majority of its ATP requirements (approximately 95%) being derived from mitochondrial oxidative phosphorylation (OXPHOS) [22]. The mitochondria, organelles that occupy approximately one-third of the adult cardiomyocyte volume, are often referred to as the cell’s ‘energy supply’ or ‘power station’ [23]. Unlike other cardiovascular diseases, heart failure has been described as a disease in which the heart acts as an ‘energy-poor engine’ [24], with common features including mitochondrial dysfunction and oxidative stress [25]. Importantly, ATP is in short supply in heart failure, and the key reason for ATP deficiency is low NAD^+^ levels [26]. NAD^+^ is the centre of OXPHOS and protein acetylation, and the NAD^+^/NADH balance is critical for mitochondrial OXPHOS [27]. Therefore, increasing NAD^+^ levels is considered a promising strategy for the treatment of heart failure. It may alleviate heart failure by ameliorating the imbalance in energy homeostasis caused by mitochondrial dysfunction [8,26,28]. However, the mechanisms by which NAD^+^ affects mitochondrial dysfunction remain to be elucidated. This study aims to provide a comprehensive summary of the potential mechanisms and current clinical therapeutic measures by which NAD^+^ regulates mitochondrial dysfunction in heart failure, with the goal of identifying new targets for the treatment of heart failure.

## 2. Materials and Methods

We used keywords to search the literature in the PubMed electronic database (“Heart Failure” [Title/Abstract] OR “Cardiac Dysfunction” [Title/Abstract]) AND (“Mitochondrial Dysfunction” [Title/Abstract] OR “Mitochondrial Damage” [Title/Abstract]) AND (“NAD^+^” [Title/Abstract] OR “Nicotinamide Adenine Dinucleotide” [Title/Abstract] OR “NAD^+^ Metabolism” [Title/Abstract]).

The search was limited to English-language papers published before February 2025.

## 3. Heart Failure

Heart failure is a leading cause of morbidity and mortality worldwide [29]. It is characterised primarily by congestion of the corporeal and pulmonary circulation due to water and salt retention, which eventually leads to symptoms such as exertional dyspnoea and signs such as peripheral oedema [30,31]. Concurrently, the heart’s capacity to function as a pump is compromised, resulting in an inability to deliver adequate blood to meet the body’s oxygen and nutritional demands [32]. The guidelines utilise ejection fraction (EF) to classify heart failure into three categories: heart failure with preserved ejection fraction (HFpEF), characterised by a left ventricular ejection fraction (LVEF) ≥ 50%; heart failure with intermediate ejection fraction (HFmrEF), with an LVEF of 41–49%; and heart failure with reduced ejection fraction (HFrEF), with an LVEF < 40%. Among them, HFpEF, which is considered to be closely related to genetic, age, and lifestyle factors, is a typical geriatric syndrome that is most common in women [33,34], and is usually associated with chronic comorbidities such as type 2 diabetes mellitus (T2DM), obesity, and arterial hypertension, which activate inflammatory responses and produce pathological changes such as hypertrophy, fibrosis, and pathological changes such as amyloidosis [35,36]. Patients with HFmrEF account for approximately 13–24% of all heart failure patients and are an intermediate type of heart failure between HFpEF and HFrEF, which is thought to be an interaction between myocardial stretch and inflammation [37,38,39]. HFmrEF is often complicated by hypertension, diabetes mellitus, and renal insufficiency [40] and is similar to HFrEF in terms of the high prevalence of ischemic heart disease [41]. HFrEF is caused by oxidative stress due to various factors, such as ischaemia, infections and viruses, leading to cardiomyocyte death, decreased pumping capacity of the heart and reduced cardiac output [42,43,44]. The common causes of HFrEF include coronary artery disease and cardiac valvular disease [35]. Activation of the renin—angiotensin—aldosterone system is the main mechanism of HFrEF, and inhibition of its overexpression is the mainstay of therapy for such patients [44]. The prevalence of HFpEF has been reported to exceed the number of patients with HFrEF in recent years [45,46,47]. However, in terms of therapeutic outcomes, patients with HFrEF respond better to pharmacological treatment regimens in terms of efficacy and prognosis [48].

The substantial amount of ATP necessary to sustain cardiac contractile function in adults is predominantly derived from mitochondrial oxidative phosphorylation and glycolysis [49]. In the normal adult heart, 95% of ATP is supplied by mitochondrial oxidative phosphorylation, with the remainder supplied by glycolysis [50,51]. It has been demonstrated that this process concomitantly results in a decrease in NAD^+^ levels [52]. The continuous production of ATP by the heart is essential for maintaining its contractile function, and if the ATP supply is not replenished in a timely manner, the heart will deplete its ATP reserves within 2–10 s, which, in turn, leads to contractile failure [53,54]. Consequently, mitochondrial energy metabolism is imperative for sustaining normal physiological activity in vivo, accounting for approximately 25% of the human cardiomyocyte volume, with the highest levels of all tissues [55,56,57], and is predominantly located in the submyocardial, perinuclear, and intrafibrillar regions of cardiomyocytes [55]. During heart failure, mitochondria undergo significant structural and functional changes. Structural alterations include mitochondrial swelling, thickening of cristae, a reduction in the number of cristae, and a decrease in the number and volume of mitochondria [58]. These changes are accompanied by impaired oxidative respiration and a substantial decrease in ATP levels [59,60]. In normal cardiomyocytes, for example, fatty acids are the primary metabolic substrate for energy supply and are supplemented by glucose, amino acids, and lactate [61]. Previous studies have shown that ATP levels in cardiomyocytes decrease by 30–40% in the heart failure state [62]. To maintain adequate ATP levels, the main source of energy metabolism in heart failure gradually shifts from fatty acids to glucose [63]. However, this transition results in a reduction in the production of energy, leading to a state of energy deprivation within the heart [64]. Additionally, the process of glycolysis generates lactate and ions, which are byproducts of metabolism and accumulate within cardiomyocytes, resulting in an overload of intracellular Na^+^ and Ca^2+^ [65]. This, in turn, causes mitochondrial dysfunction and endoplasmic reticulum stress, further exacerbating myocardial injury [65]. Mitochondrial dysfunction has been shown to promote the generation of large amounts of reactive oxygen species (ROS) [66,67], and excessive accumulation of ROS has been demonstrated to cause damage to mitochondrial DNA and mitochondrial proteins, which in turn exacerbates mitochondrial dysfunction and activates mitochondria-mediated apoptosis of cardiomyocytes, resulting in a vicious cycle [68], which ultimately leads to the exacerbation of heart failure.

An increasing number of studies have shown that mitochondrial dysfunction, including abnormal mitochondrial dynamics, mitochondrial oxidative stress damage (ROS overproduction), abnormal mitochondrial autophagy, and dysregulated mitochondrial Ca^2+^ homeostasis, is closely related to the development of heart failure [69]. Therefore, targeting mitochondrial dysfunction may be a potentially effective strategy for the treatment of heart failure.

### 3.1. Mitochondria-Mediated Pathology of Heart Failure

#### 3.1.1. Heart Failure and Mitochondrial Dynamics

As highly dynamic organelles, mitochondria undergo rapid and continuous processes of fission and fusion when the cell is subjected to environmental stress or has a need for energy metabolism. This process maintains the homeostasis of the network structure, known as mitochondrial dynamics [70,71,72]. The process of mitochondrial dynamics involves two primary mechanisms: mitochondrial fusion and mitochondrial fission. The process of mitochondrial fusion is mediated by dynamin-associated GTPase—mitofusins 1 (MFN1), MFN2, and optic atrophy 1 protein (OPA1) [73]. MFN1 and MFN2 promote the fusion of the outer mitochondrial membrane (OMM), whereas OPA1 is responsible for promoting the fusion of the inner mitochondrial membrane (IMM) [74]. Mitochondrial fusion has been demonstrated to occur in a manner that involves the amalgamation of intact and moderately dysfunctional mitochondria, thereby preserving the integrity of the organelle and facilitating the exchange of substances between substrates, mitochondrial DNA, and metabolites. This process is believed to contribute to the restoration of function in defective mitochondria [75]. Mitochondrial fission is governed primarily by dynamin-related protein 1 (Drp1) [76]. In addition, numerous other proteins that target the outer membrane, including mitochondrial fission protein 1 (Fis1) and mitochondrial fission factor (Mff), play a role in mitochondrial fragmentation [70]. The process of mitochondrial fission removes functionally impaired mitochondria from the cell, thereby facilitating mitochondrial transport and ensuring control over the mitochondrial mass [77]. A dynamic balance between mitochondrial fusion and fission is essential for maintaining mitochondrial homeostasis and ensuring optimal cardiac function [78].

A hallmark of heart failure is the dysregulation of mitochondrial dynamics [79]. Numerous studies have demonstrated that OPA1 and MFN2 expression are reduced and that Drp1 levels are increased in patients with heart failure [80]. The systemic expression of OPA1 is essential for enhancing mitochondrial cristae stability and mitochondrial respiratory function, increasing myocardial fatty acid utilisation and decreasing ROS production, effectively attenuating mitochondrial dysfunction to maintain mitochondrial morphology during heart failure and preventing apoptosis [81]. Conversely, the knockdown of OPA1 results in mitochondrial breakage, the induction of abnormal mitochondrial morphology and cardiac dilatation, and a reduction in cardiomyocyte contractility [82,83]. Moreover, this has been shown to increase the risk of cardiomyopathy [84]. Similarly, the deletion of MFN1 and MFN2 contributes to the development of cardiac hypertrophy [85]. Drp1 is a key regulator of mitochondrial division and is normally located in the cytoplasm. It is recruited into the mitochondria under conditions of stress and is capable of responding to energy stress and regulating mitochondrial autophagy [86]. Increased Drp1 activity promotes mitochondrial fission [76]. In heart failure, excessive mitochondrial fission has been demonstrated to cause a reduction in mitochondrial mass and dysfunction, thus disrupting mitochondrial homeostasis and leading to apoptosis and myocardial injury [87]. Consequently, aberrant mitochondrial dynamics result in mitochondrial dysfunction, thereby disrupting energy metabolism and ultimately leading to heart failure.

#### 3.1.2. Heart Failure and Mitochondrial Autophagy

Mitochondrial autophagy represents a pivotal mitochondrial quality control mechanism in cardiomyocytes, whereby damaged or dysfunctional mitochondria are selectively eliminated to ensure the maintenance of mitochondrial homeostasis and promote cell survival [88,89,90]. Under normal conditions, mitochondrial autophagy exerts a protective effect on cardiomyocytes, and the increase in autophagy in heart failure improves mitochondrial function and reduces cardiomyocyte apoptosis. Conversely, insufficient autophagy exacerbates the development of heart failure [91]. Abnormal mitochondrial autophagy has been shown to lead to an increase in defective mitochondria, which are unable to produce ATP and other biosynthetic products. In addition, these defective mitochondria release large amounts of ROS [92]. The excessive production of ROS in the mitochondria has been shown to induce apoptosis and reduce the antioxidant capacity of cells, thereby impairing the oxidative function of mitochondrial DNA and proteins and promoting myocardial remodelling and fibrosis [93]. Some damaged mitochondria, which cannot be completely degraded by autophagy, accumulate in cardiomyocytes, vascular smooth muscle cells (VSMCs), and endothelial cells, triggering an inflammatory response and inducing myocarditis and dilated cardiomyopathy [94]. These myocardial diseases lead to varying degrees of myocardial fibrosis and hypertrophy, further exacerbating the pathological process of heart failure [95,96].

PTEN-induced kinase 1/Parkin RBR E3 ubiquitin-protein ligase (Pink1/Parkin) is thought to be an important component that mediates mitochondrial autophagy in cardiomyocytes and is intimately involved in heart failure [97]. Pink1 and Parkin have a direct functional link, and Pink1 is an upstream protein of Parkin that recruits Parkin to maintain normal mitochondrial function in cells [98]. Studies have shown that defects in Pink1/Parkin-mediated mitochondrial autophagy adversely affect the heart. For example, Pink1 protein levels are significantly reduced in human end-stage heart failure, and Parkin deficiency leads to the accumulation of dysfunctional mitochondria in cardiomyocytes, the development of heart failure, and, ultimately, an increase in myocardial infarction size in mice [99,100]. In addition, Pink1 deficiency hinders Parkin translocation to mitochondria and attenuates mitochondrial autophagy, which leads to increased cardiomyocyte apoptosis accompanied by fibrosis and reduced capillary density, all of which are closely associated with the clinical manifestations of heart failure [99]. Therefore, Pink1/Parkin-mediated mitochondrial autophagy plays an important role in promoting cardiomyocyte survival and could be a potential strategy for the treatment of heart failure.

#### 3.1.3. Heart Failure and Mitochondrial Oxidative Stress

Oxidative stress is defined as an imbalance between the production of ROS and the cellular antioxidant defence system, resulting in impaired redox signalling and/or molecular damage [101,102]. Mitochondria represent a significant source of ROS production within cells [103]. Excessive ROS production in cardiomyocytes typically occurs during periods of mitochondrial dysfunction, which in turn causes irreversible damage to the mitochondria, ultimately contributing to the development of cardiovascular disease [104]. The heart is a major source of ROS, the majority of which are derived from mitochondria, nicotinamide adenine dinucleotide phosphate (NADPH) oxidase, xanthine oxidase, and endothelial nitric oxide synthase (eNOS) [102,105]. In heart failure, mitochondrial respiratory chain complex I produces large amounts of negative superoxide ions (O^2−^), leading to uncoupling of the heart failure mitochondrial respiratory chain and exacerbating ROS production [102,106]. NADPH oxidase can be activated by a wide range of factors associated with cardiovascular pathology, including tumour necrosis factor-alpha (TNF-α), noradrenaline, angiotensin II, and mechanical tension, thereby increasing ROS levels [107,108,109]. Xanthine oxidase (XO) activity has been shown to increase in the failing human heart, leading to oxidative stress and causing dysregulation of myocardial function and energy expenditure [110]. Furthermore, eNOS uncoupling has been demonstrated to increase myocardial ROS expression, leading to dilatory remodelling and cardiac insufficiency [111].

It has been demonstrated that, in heart failure, there is not only an excess of ROS but also a decrease in the activity of antioxidant enzymes. For example, the activities of antioxidant enzymes, including superoxide dismutase, glutathione peroxidase (GSH-Px), and catalase, are markedly diminished in heart failure [112,113,114]. The proinflammatory response is a hallmark of heart failure, and the overproduction of inflammatory factors (e.g., TNF-α and interleukin-6 (IL-6)) can induce mitochondrial DNA damage, inhibit antioxidant factors and promote ROS generation [115]. Increased ROS activate matrix metalloproteinases (MMPs), which, in turn, induce cardiomyocyte apoptosis through the mitogen-activated protein kinase (MAPK) and nuclear factor kappa-B (NF-κB) pathways, accompanied by the proliferation of myocardial fibroblasts, thereby promoting myocardial fibrogenesis [116]. Furthermore, substantial amounts of ROS directly interact with intracellular macromolecules, including proteins, lipids, and DNA, resulting in cardiomyocyte membrane damage, cardiomyocyte dysfunction, and the progression of heart failure [102]. Consequently, mitochondrial oxidative stress has the capacity to induce cardiomyocyte apoptosis and promote the progression of heart failure.

#### 3.1.4. Heart Failure and Mitochondrial Ca^2+^ Homeostasis

Ca^2+^ is a ubiquitous second messenger in cells that regulates a variety of biological functions, including cardiac contraction, muscle contraction, synaptic transmission, cell proliferation, and apoptosis [117]. Dysfunction of excitation—contraction coupling [118], an essential process that regulates cardiac systolic and diastolic functions, is one of the common pathophysiological manifestations of cardiac remodelling [119]. As a pivotal factor regulating ECC, dysregulation of Ca^2+^ homeostasis has been shown to result in myocardial contractile dysfunction and the onset of arrhythmias, in addition to its interference with energy metabolism and its impact on cell survival and mitochondrial function [120]. This has led to its classification as a major cause of death in patients with heart failure. Research has demonstrated that the onset of defective ECC function in heart failure patients is closely associated with the dysfunction of receptors, ion pumps, and related regulatory proteins involved in Ca^2+^ cycling [121]. Furthermore, Ca^2+^ is imperative for ATP production, which in turn transmits energy requirements to the mitochondria [122,123]. Conversely, aberrant mitochondrial energy metabolism in heart failure has been demonstrated to disrupt Ca^2+^ homeostasis, thereby instigating a detrimental cycle that culminates in more profound myocardial injury [124].

**Figure 2:**
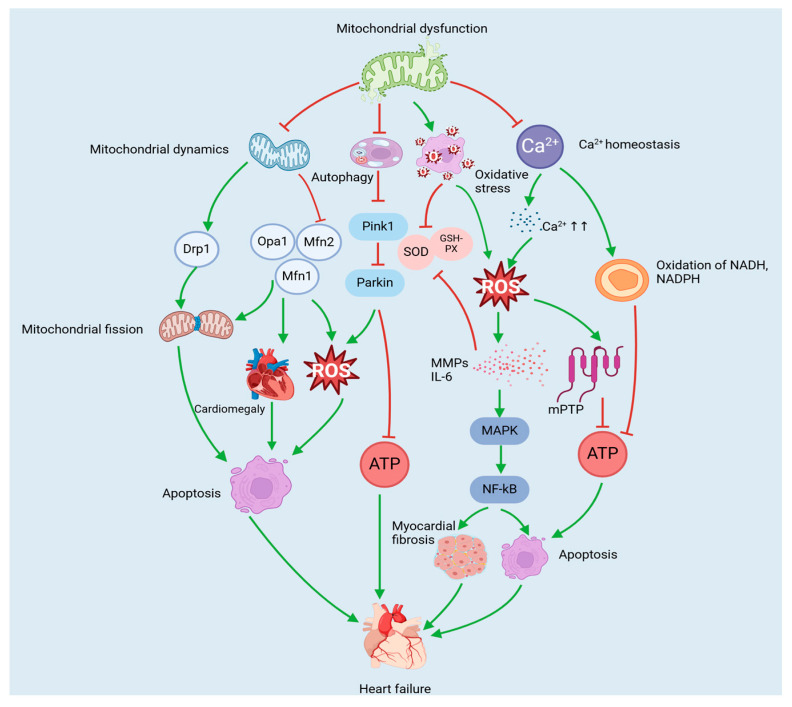
Mitochondria-mediated pathology of heart failure. Mitochondrial dysfunction leads to abnormal mitochondrial dynamics, oxidative stress injury, dysfunctional mitochondrial autophagy, and disturbed Ca^2+^ homeostasis. Together, these abnormalities contribute to the development of heart failure by inhibiting ATP production and inducing cardiomyocyte apoptosis, cardiac hypertrophy, and myocardial fibrosis.

Ca^2+^ overload has the capacity to disrupt mitochondrial physiology [125]. For example, an overaccumulation of Ca^2+^ within the mitochondrial matrix can result in mitochondrial swelling, rupture of the outer membrane, and the release of ‘apoptotic sources’ (e.g., cytochrome c contained in the intermembrane space) into the external medium and stimulate ROS production, inducing a sustained opening of the mitochondrial permeability transition pore (mPTP) in the IMM. The inhibition of ATP production promotes cardiomyocyte apoptosis, which results in mitochondrial damage and myocardial remodelling, ultimately leading to heart failure [125,126]. An imbalance between Ca^2+^ and ATP also leads to the oxidation of pyridine nucleotides (e.g., NADH and NADPH) in the mitochondrial matrix. The oxidation of NADH has been demonstrated to reduce the efficiency of ATP synthesis by interfering with the electron transport chain, thereby impairing the contractile function of cardiomyocytes. In contrast, the oxidation of NADPH has been shown to deplete the cellular antioxidant reserve and trigger oxidative stress injury, ultimately leading to maladaptive cardiac remodelling and further exacerbating the progression of heart failure [127]. In addition, the mitochondrial Ca^2+^ content is critical for the regulation of mitochondrial autophagy [128,129]. AMP-activated protein kinase (AMPK) is a highly conserved energy-sensing kinase that responds to activation in response to reduced ATP [130]. The inhibition of mitochondrial Ca^2+^ uptake has been shown to increase AMPK activity and activate mitochondrial autophagy via the AMPK/mammalian target of rapamycin (mTOR)/Unc-51-like kinase 1 (ULK1) signalling pathway [131,132], thereby promoting a protective effect against cardiac effects during the progression of heart failure. Consequently, Ca^2+^ levels are critically important for cardiomyocytes and can influence heart failure by regulating mitochondrial function (Figure 2, Table 1).

### 3.2. Effects of Other Metabolic Diseases on Heart Failure

Metabolic diseases, including obesity, insulin resistance, and type 2 diabetes mellitus, can affect heart failure in a NAD^+^-dependent and independent manner. Obesity is associated with an increased risk of cardiovascular disease (CVD), particularly with the development of heart failure. For example, the renin-angiotensin-aldosterone system (RAAS) and the sympathetic nervous system are simultaneously activated in obesity, leading to cardiac remodelling and left ventricular hypertrophy (LVH), which promotes the development of heart failure [133]. In addition, proinflammatory cytokines (TNF-α, IL-1β, etc.) produced by adipose tissue have been associated with a poor prognosis of heart failure [134]. Choi et al. [135] discovered that in obese mice, elevated hepatic microRNA-34a suppressed the expression of nicotinamide phosphoribosyltransferase (NAMPT) and silent information regulator 1 (SIRT1), which led to a decrease in NAD^+^ levels and SIRT1 deacetylase activity, inducing cardiovascular metabolic diseases. Heart failure is one of the most common complications of diabetes mellitus, while diabetes mellitus is also an independent risk factor for heart failure (including coronary artery disease, insulin use and elevated serum creatinine in diabetic patients) [136]. In particular, insulin resistance, a hallmark of T2DM, can also develop from heart failure through oxidative stress dependent on NAD(P)H oxidase, which triggers dysfunction of insulin signalling in skeletal muscle [137]. Studies have shown that in diabetic patients, persistent hyperglycemic state and insulin resistance may contribute to the development of heart failure through mechanisms such as activation of the sympathetic nervous system and exacerbation of abnormalities in cardiovascular function [138]. Chiao et al. [139] found that the redox imbalance of mitochondrial NAD^+^/NADH increases the risk of heart failure and leads to diabetic cardiomyopathy. Furthermore, the supplementation of NMN has been shown to enhance NAD^+^ biosynthesis and SIRT1 activity, which may contribute to the improvement of T2DM [140]. In summary, metabolic diseases such as obesity and diabetes can exacerbate the development of heart failure independently or in an NAD^+^-dependent manner.

## 4. NAD^+^ and Mitochondrial Biogenesis

Mitochondrial biosynthesis is a complex biological process involving the growth and division of mitochondria and the formation of new organelle structures. It also coordinates the synthesis of proteins jointly encoded by nuclear and mitochondrial DNA (mtDNA) [141]. As a key regulator of mitochondrial homeostasis, SIRT1 is a deacetylase that promotes mitochondrial biosynthesis by activating peroxisome proliferator-activated receptor γ coactivator 1α (PGC-1α) activity through deacetylation [142]. Conversely, SIRT1 is an important NAD^+^-dependent deacetylase whose activation is highly dependent on intracellular NAD^+^ levels. Therefore, the concentration of intracellular NAD^+^ directly regulates the efficiency of mitochondrial biosynthesis mediated by PGC-1α by modulating the deacetylase activity of SIRT1. Additionally, overexpression of the NAD^+^-dependent deacetylase SIRT3 promotes the increased expression of mitochondrial biosynthesis genes and proteins (PGC-1α and TFAM) to further maintain mitochondrial function and health [143]. Furthermore, exogenous supplementation of NAD^+^ precursors (e.g., nicotinamide riboside (NR)) has been shown to enhance mitochondrial oxidative metabolism and biosynthesis, prevent mitochondrial ultrastructural abnormalities and mtDNA deletion formation, and reverse mitochondrial dysfunction in ageing or disease states [144]. In summary, NAD^+^ is essential for mitochondrial biosynthesis, and its elevated levels are beneficial for enhancing mitochondrial function and delaying the onset of ageing-related diseases through activation of the Sirtuins family and other mechanisms.

## 5. NAD^+^ Modulates Mitochondria to Improve Heart Failure

Low levels of NAD^+^ have been identified as a significant factor contributing to the suboptimal supply of ATP in heart failure [26]. The imbalance of NAD^+^ in cardiomyocytes is a direct cause of metabolic remodelling and mitochondrial dysfunction in failing hearts. Conversely, supplementation with NAD^+^ precursors effectively preserves the ultrastructure of mitochondria and enhances mitochondrial fatty acid oxidation (FAO) while concomitantly impeding the increase in ROS and preventing cardiomyocyte apoptosis [27]. Furthermore, elevated levels of NAD^+^ promote the normalisation of mitochondrial protein acetylation, thus contributing to the improvement of heart failure [27]. Consequently, the maintenance of stable intracellular NAD^+^ levels is regarded as a promising heart failure treatment that can enhance cardiomyocyte bioenergetics and overall cardiac function by improving mitochondrial dysfunction [26,52].

Sirtuins are a group of highly conserved NAD^+^-dependent deacetylases. An increase in NAD^+^ levels enhances the activity of sirtuin proteins, which significantly inhibits myocardial hyperacetylation and improves myocardial mitochondrial function. In turn, this impacts cellular metabolism and homeostasis [145,146]. SIRT1, a particularly well-studied sirtuin, is found in the cell nucleus and regulates biological processes such as cellular autophagy and differentiation through the deacetylation of various histones and nonhistone proteins [147,148]. NAD^+^ has been shown to enhance endothelial cells by activating the SIRT1-induced release of vascular endothelial growth factor (VEGF) and basic fibroblast growth factor (FGF) from myocytes, thereby effectively preventing vascular ageing and reducing the risk factors for heart failure [149]. In addition, NAD^+^ has been shown to promote energy metabolism in cardiomyocytes by regulating mitochondrial production and function through the SIRT1/proliferator-activated receptor γ coactivator-1α (PGC1α) pathway. Concurrently, AMPK enhances SIRT1 activity by increasing NAD^+^ levels in cells, thereby regulating energy expenditure [150]. Persistent cardiac hypertrophy can ultimately result in heart failure [151,152]. Consequently, the inhibition of cardiac hypertrophy may impede the progression of heart failure and increase patient survival [153]. SIRT3 is located in the mitochondrial matrix and is the major mitochondrial deacetylase [154]. In the mitochondrial electron transport chain, SIRT3 directly binds to and regulates succinate dehydrogenase A and ATP synthase (complex V) of complex I and complex II, thereby effectively increasing ATP levels [155]. NAD^+^ has been shown to prevent cardiac hypertrophy and alleviate heart failure [156,157]. Isocitric dehydrogenase 2 (IDH2) is the main antioxidant enzyme in mitochondria and the main source of NADPH [158]. SIRT3 increases the activities of superoxide dismutase 2 (SOD2) and IDH2 to reduce ROS generation and reduce oxidative stress [159,160]. In addition, NAD^+^ has been demonstrated to stabilise Ca^2+^ homeostasis in mitochondria, regulate mitochondrial calcium uniporter (MCU) complex function through SIRT3, and prevent Ca^2+^ overload-induced mPTP opening, which ultimately attenuates cardiomyocyte apoptosis or necrosis [161,162].

NAD^+^ and its reduced form, NADH, are involved in the redox system, which is the main biological oxidation mechanism for the electron transfer process in the respiratory chain and is essential for the completion of oxidative reactions [163]. NAD^+^ redox imbalance has been demonstrated to be a key mediator of pressure overload-induced heart failure [157]. ROS (e.g., superoxide anion O^2−•^ and nitric oxide radical NO^•^) are important signalling molecules involved in the regulation of biological responses, and an imbalance in their homeostasis is associated with cellular dysfunction and disease occurrence is closely related [20]. In pathological conditions, excessive generation of ROS disrupts the cellular antioxidant defence system, leading to decreased free radical scavenging capacity, creating a state of oxidative stress and ultimately triggering abnormal cardiac function [20]. NAD^+^ supplementation (e.g., NAM) can act as an O^2−•^ radical scavenger and significantly enhance intracellular NAD^+^ content and ATP synthesis efficiency by enhancing the protein expression levels of superoxide dismutase and catalase. Additionally, it has been observed to reduce mitochondrial ROS generation, inhibit the loss of mitochondrial membrane potential, and ultimately exert an antioxidant protective effect on cardiomyocytes [164].

Research has demonstrated that a diminished NAD^+^/NADH ratio is indicative of mitochondrial dysfunction in heart failure [157] and that increasing the NAD^+^ content effectively ameliorates mitochondrial redox processes, restores the NAD^+^/NADH ratio, and ultimately mitigates heart failure [21]. NMN is a precursor of NAD^+^ that enhances metabolic flux and reduces cellular reactive oxygen species production through the TCA cycle and electron transport chain [165]. The protective effect of NMN supplementation on cardiac function has been demonstrated by the augmentation of NAD^+^ levels, which has been shown to reduce vascular oxidative stress and improve vascular and mitochondrial dysfunction [166]. Furthermore, NMN has been demonstrated to increase SOD2 activity by facilitating SIRT3-dependent deacetylation while concomitantly reducing ROS production within mitochondria and thereby maintaining cardiomyocyte homeostasis [167]. Nicotinamide riboside, a dietary source of NAD^+^ [168], is metabolised to NMN in cells via the phosphorylation of nicotinamide riboside kinase (NRK), which has been demonstrated to increase both the cellular and the mitochondrial NAD^+^ content [169]. Koay et al. [170] demonstrated that NR therapy enhances myocardial antioxidant capacity by increasing NADPH levels and promoting the regeneration of reduced glutathione in HFpEF hearts, thereby preventing cardiomyocyte damage induced by ROS. Furthermore, NR supplementation has been shown to enhance cardiac function by increasing myocardial NAD^+^ levels and promoting MFN2-mediated mitochondrial fusion through the modulation of the SIRT1-PGC1α-peroxisome proliferator-activated receptor-α (PPARα) axis [171]. Insulin/insulin-like growth factor-1 (IGF-1) is a key nutrient-sensing and inhibitory pathway for cardiac autophagy, and studies have shown that elevated NAD^+^ levels can restore cardiac autophagy levels by inhibiting the activity of the IGF-1 signalling pathway, thereby alleviating the pathological process of HFpEF [172].

In addition, myocardial ischaemia is a common risk factor for heart failure. The malate-aspartate shuttle (MAS), a redox shuttle that transports reducing equivalents across the inner mitochondrial membrane, transfers NADH to the mitochondrial matrix, and maintains redox homeostasis by regenerating NAD^+^, is the primary shuttle mechanism in the heart [173]. During complete myocardial ischaemia, glycolysis becomes the major source of ATP. Whereas MAS is critical for the maintenance of glycolysis rate, it recycles NADH generated from glycolysis to cytoplasmic NAD^+^ via mitochondria to maintain glycolytic activity [174]. It has been reported that blockade of the MAS pathway by application of amino acid aminotransferase inhibitor aminooxyacetic acid (AOA) during myocardial ischaemia and the early phase of reperfusion can produce cardioprotective effects similar to ischemic preconditioning (IPC), resulting in reduction of myocardial infarct size and improvement of hemodynamic response after reperfusion [175]. Pyruvate dehydrogenase (PDH) is the rate-limiting enzyme for glucose oxidation in the heart, converting pyruvate to acetyl coenzyme A, which plays a key role in energy metabolism [176]. In ischaemia-induced heart failure, the ratio of NADH to NAD^+^ is increased, and elevated NADH activates PDH kinase isoforms, which inhibit PDH and glucose oxidation, further reducing acetyl coenzyme A production [177]. Ischemic preconditioning reduces NADH levels and restores NAD^+^/NADH balance by activating the AMPK and SIRT1 pathways and inhibiting PDH kinase isoform activity, activating PDH and ultimately increasing the conversion of pyruvate to acetyl coenzyme A [177,178,179]. In addition, hemorrhagic shock occurs as a result of rapid blood loss, characterised by cellular hypoxia, which can lead to myocardial ischaemia [180]. During cellular hypoxic metabolism, a large accumulation of lactic acid can be generated, which can easily induce metabolic acidosis [180]. Studies have shown that supplementation of the NAD^+^ precursor NMN can promote the balance of the NAD^+^/NADH ratio and reduce the conversion of pyruvate to lactate via lactate dehydrogenase (LDHA), thereby decreasing the level of lactate in the myocardium and inhibiting metabolic acidosis [180,181].

**Figure 3:**
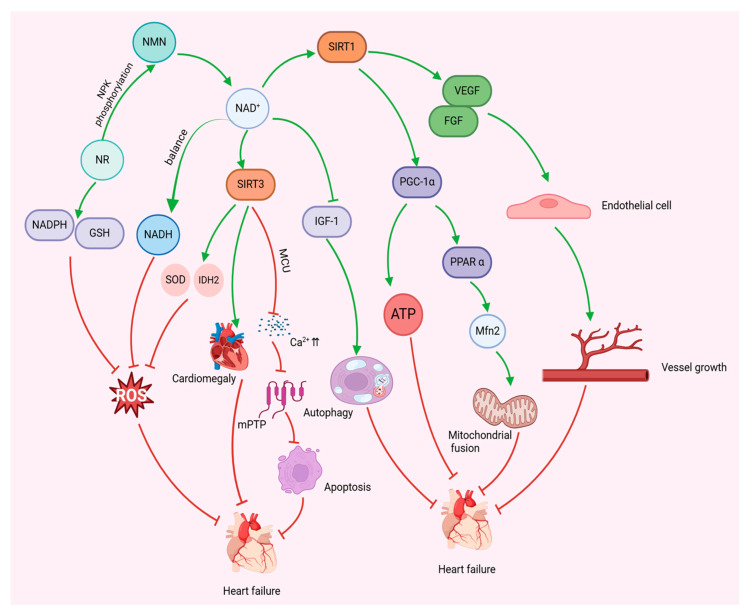
NAD^+^ modulates mitochondria to improve heart failure. NAD^+^ regulates mitochondrial biosynthesis and anti-oxidative stress, inhibits cardiac hypertrophy, regulates mitochondrial autophagy and fusion, and promotes angiogenesis through activation of SIRT1 and SIRT3, thereby reducing cardiomyocyte apoptosis, restoring myocardial energy metabolism, and ultimately improving heart failure.

Taken together, these findings suggest that NAD^+^ may have cardioprotective effects against heart failure through multiple mechanisms, including the inhibition of myocardial hyperacetylation, the prevention of mitochondrial Ca^2+^ overload, the reduction in mitochondrial oxidative stress, and the modulation of mitochondrial autophagy and fusion. These findings suggest that NAD^+^-enhancing interventions may be a potential strategy for the treatment of heart failure (Figure 3).

### 5.1. Increased Levels of NAD^+^

To date, strategies to increase NAD^+^ levels fall into two categories: the inhibition of NAD^+^ consumption and the supplementation of NAD^+^ precursors to promote NAD^+^ biosynthesis [182].

### 5.2. NAD^+^ Precursor Supplementation

NAD^+^ can be produced via the remedial pathway through NR, NMN, NAM, or NA. NR, NAM, and NA, which are collectively known as the three different forms of vitamin B3, have demonstrated the potential to be used as dietary supplements to increase the intracellular levels of NAD^+^ [182].

#### 5.2.1. NR

NR is a naturally occurring pyridine nucleoside form of vitamin B3 that is found in milk and can be taken as a dietary supplement [183]. Furthermore, NR has been identified as a precursor of the NAD^+^ salvage pathway [184]. Oral administration of NR has been demonstrated to be well tolerated without adverse effects and has been shown to increase NAD^+^ pools in the nucleus, cytoplasm, and mitochondria [183,184]. In addition, NR has been shown to be cardioprotective in a variety of mouse models of cardiomyopathy [8,185]. For example, in a mouse HFpEF model, NR supplementation has been shown to inhibit FAO enzyme hyperacetylation, promote normalisation of the NAD^+^/NADH ratio, alleviate diastolic dysfunction, and restore mitochondrial function, thereby improving the signs and functional performance of HFpEF patients [186,187]. As an electron donor, NADH produced by the TCA cycle plays an important role in the synthesis of ATP via oxidative phosphorylation [170]. NR supplementation increases the levels of three rate-limiting TCA enzymes used for NADH production, including malate dehydrogenase 1 and 2 (MDH1, MDH2) and IDH3, which leads to an increase in mitochondrial ATP production, modulation of myocardial energy expenditure, and ultimately an improvement in heart failure in HFepEF mice [170].

During the development of heart failure, increased mitochondrial damage associated with increased oxidative stress and impaired quality control mediated by mitochondrial autophagy induces an inflammatory response in the failing heart [188,189]. Specifically, it is hypothesised that excessive accumulation of ROS produced by mitochondria plays a pivotal role in activating the secretion of proinflammatory cytokines [190]. NR treatment has been shown to reduce mitochondrial ROS production and inhibit the expression of proinflammatory factors, such as NLRP3 inflammatory vesicles and IL-1β and IL-18, as well as enhance mitochondrial respiration, which is beneficial for patients with heart failure [191,192]. Consequently, NR has been identified as a promising therapeutic agent for the treatment of heart failure, given its ability to modulate mitochondrial dysfunction.

#### 5.2.2. NMN

NMN is an intermediate product of NAD^+^ biosynthesis. It can be derived from the phosphorylation of NR or generated by the conversion of ribose-5-phosphate with NAM [169,193]. It has been identified in a variety of natural foods, including snap peas, tomatoes, broccoli, milk, avocados and raw beef [194,195]. The administration of NMN has been shown to increase the production of NMN by replenishing mitochondrial NAD^+^ pools, thereby enhancing cellular metabolism and promoting ATP production [27]. Notably, NMN upregulates the mRNA expression level of nicotinamide mononucleotide adenylyltransferase (NMNAT), a central enzyme in NAD^+^ biosynthesis, thereby increasing the NAD^+^ content [196]. The protective effect of NMN against heart failure has been demonstrated in several studies [197,198]. For example, Zhang et al. [27] demonstrated that short-term administration of NMN protects mice from stress overload-induced heart failure by reducing the amount of ROS in the mitochondria, maintaining mitochondrial homeostasis, and protecting cardiomyocytes from stress-induced apoptosis. Long-chain acyl-CoA dehydrogenase (LCAD) is a FAO enzyme, and Sirt3 regulates mitochondrial fatty acid metabolism by mediating the enzymatic activity of LCAD. In addition, NMN treatment has been shown to induce mitochondrial FAO expression, suggesting that NMN improves cardiac function and cardiac energy [140,199].

An elevated NADH/NAD^+^ ratio (i.e., NAD^+^ redox imbalance) induces mitochondrial protein hyperacetylation, which makes the heart susceptible to pressure overload and increases the risk of developing heart failure [157]. NMN normalises the NAD^+^/NADH ratio, restores the NAD^+^ redox balance, and reduces protein hyperacetylation, thereby attenuating the pathological manifestations and development of heart failure [157]. In addition, under normal conditions, the majority of cardiac ATP requirements are met by the oxidation of mitochondrial β-fatty acids [118]. ATP associated with glycolysis is commonly associated with cardiac pathology and heart failure [200,201]. However, some studies have shown that glycolysis is associated with cardioprotection [202]. NMN may induce a protective effect on the heart during ischaemia by stimulating glycolytic metabolism to increase ATP synthesis [202]. In summary, NMN inhibits mitochondrial oxidative stress and promotes ATP production to protect against heart failure.

#### 5.2.3. NAM

NAM, an amide form of vitamin B3, is a component of coenzyme I and coenzyme II, which are involved in cellular respiration and energy synthesis [203,204]. The NAM salvage synthesis pathway occurs via the precursor NAM or catalytically via NAMPT synthesis of NMN to produce NAD^+^ [205]. NAM promotes the protein expression of intracellular superoxide dismutase and catalase, thereby increasing the intracellular NAD^+^ content and ATP levels, decreasing the abundance of mitochondrial ROS, and preventing the loss of the mitochondrial membrane potential, ultimately ameliorating cardiomyocyte apoptosis and cell death [164]. Diastolic dysfunction is a clinical hallmark of HFpEF [206]. Abdellatif et al. [207] reported that NAM supplementation increased the levels of NAD^+^ and NAM in the heart and liver and increased plasma NAM in ZSF1 obese rats, which resulted in a significant improvement in the cardiac phenotype and alleviation of diastolic dysfunction in the myocardium. Furthermore, NAM has been shown to increase the levels of several metabolites and oxidative phosphorylation-related genes associated with the TCA cycle. In addition, NAM has been shown to restore the restricted myocardial energy reserve and improve HFpEF in ZSF1 obese rats [207].

AMPK is a vital energy sensor in cells that plays a multifaceted role in various physiological processes. It is involved in the promotion of ATP production and catabolism, maintenance of intracellular energy homeostasis and metabolic balance, and regulation of mitochondrial function and biosynthesis [208]. NAM has been shown to significantly promote the activation of the AMPK pathway, induce intracellular ATP synthesis, and ameliorate mitochondrial oxidative stress caused by ischaemia and hypoxia in cardiomyocytes, thereby preventing injury to these cells [209]. The mTOR pathway is a downstream target of AMPK and a major regulator of cellular metabolism, and the modulation of mTOR promotes cell survival and growth [210]. Conversely, the mTOR protein functions as an upstream regulatory protein of the autophagy pathway, and the activation of mTOR can inhibit the autophagy initiation mechanism to prevent the further formation of autophagosomes [211]. Li et al. [212] reported that NAM could ameliorate myocardial injury caused by hypoxia by inhibiting the mTOR pathway to increase the level of intracellular autophagy. Consequently, NAM effectively protects cardiomyocytes by increasing mitochondrial energy production and autophagy levels.

#### 5.2.4. NA

**Figure 4:**
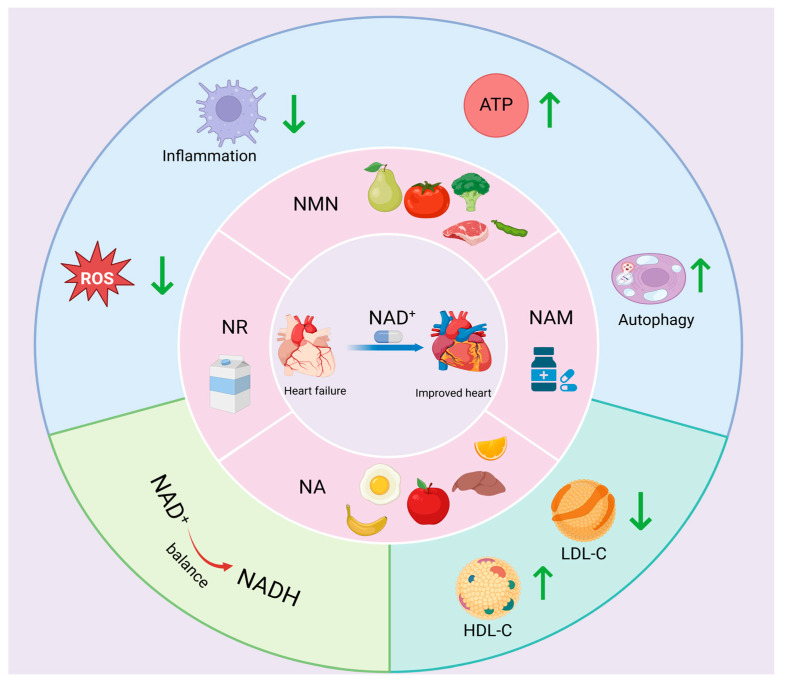
NAD^+^ precursors regulate heart failure. Myocardial NAD^+^ levels can be significantly elevated by supplementation with NAD^+^ precursors (NAM, NMN, NR, NA, etc.), which inhibit ROS generation and inflammatory responses, regulate cardiomyocyte autophagy, promote ATP synthesis and NAD^+^/NADH homeostasis, increase HDL-C content, and decrease LDL-C levels, thereby improving heart failure.

NA, also known as vitamin B3, is one of the 13 essential vitamins in the human body. NA is absorbed in the body and converted to NAM and participates in lipid metabolism, tissue respiration, and other processes as a coenzyme [213,214]. It is well known that lipoprotein(a) (Lp(a)) is an independent risk factor for CVD [215]. In contrast, NA increases high-density lipoprotein cholesterol (HDL-C) and decreases low-density lipoprotein cholesterol (LDL-C), which plays an important role in the treatment of cardiovascular diseases [216]. Epidemiological investigations have shown that higher dietary niacin intake can reduce the risk of CVD death by 37% [217], and the U.S. National Institutes of Health recommends a daily dietary allowance of niacin for adults (16 mg for men and 14 mg for adult women) [217], which is beneficial to cardiovascular health. There are few studies on the ability of NA to improve heart failure, but there are still studies that have demonstrated that NA intake significantly reduces the degree of oxidative stress in cardiomyocytes in rats with chronic heart failure, with potential cardioprotective effects [218] (Figure 4, Table 2).

#### 5.2.5. Inhibition of NAD^+^ Consumption

Intervention strategies that target NAD^+^-degrading enzymes and their regulatory pathways have been shown to have great potential for treating diseases associated with reduced NAD^+^ levels. Specifically, another method for increasing NAD^+^ is through the inhibition of poly ADP-ribose polymerase (PARP) or NADases (including the cluster of differentiation 38 (CD38), the cluster of differentiation 157 (CD157), and sterile alpha and toll/interleukin-1 receptor motif-containing (SARM)) to inhibit their degradation [205]. CD38 is one of the major NADases in mammals [219]. Inhibition of CD38 has been demonstrated to inhibit cardiac hypertrophy and prevent heart failure by activating the SIRT3-FOXO3-mediated antioxidant signalling pathway [220]. The use of CD38 inhibitors, such as lignocaine, has been demonstrated to augment NAD^+^ levels, thereby providing effective protection for cardiac endothelial and cardiomyocyte function in a murine model of myocardial ischaemia [221]. Quercetin, a natural flavonoid, has been shown to inhibit CD38 in vitro [222]. In addition, quercetin supplementation has been found to reduce some of the risk factors for cardiovascular disease and to exert cardioprotective effects [223]. Apigenin, a novel CD38 inhibitor, has been shown to increase the NAD^+^ content in a variety of tissues by inhibiting CD38 activity and can reduce the degree of protein acetylation by activating the deacetylation function of SIRT1 and SIRT3 [224,225]. This, in turn, has been shown to increase the ratio of intracellular NAD^+^/NADH metabolism. Apigenin also enhances mitochondrial antioxidant enzyme activity via SIRT3 [226], which prevents cardiovascular diseases. In addition, 78c, a potent and specific thiazoloquinolone analogue, acts as a CD38 inhibitor, which not only significantly elevates the NAD^+^ concentration in plasma, liver and muscle tissues but also effectively reduces the incidence of myocardial infarction and exerts cardioprotective effects by modulating NAD^+^ metabolism to attenuate postischemic myocardial injury [227,228]. SARM1 is another NADase. SARM1 is another NADase that, when activated by neuronal injury, causes rapid depletion of NAD^+^, resulting in a significant decrease in cellular ATP levels [229,230]. XAV939, a SARM1 inhibitor employed in the treatment of neurological disorders and axonal injury, has been shown to promote increased NAD^+^ levels and alleviate energy depletion [231]. PARP-1, a key player in the detection and repair of DNA damage, has been shown to activate PARP-1, resulting in the depletion of intracellular NAD^+^ levels by up to 80% [232]. Inhibition of PARP enhances NAD^+^ availability in tissues, improves mitochondrial function and SIRT1 activity, and ameliorates dysfunction associated with mitochondrial defects [232]. Overall, on the basis of the available studies, the therapeutic strategy of reducing aberrant intracellular NAD^+^ depletion by targeting and inhibiting the activities of NAD^+^-depleting enzymes (e.g., PARPs, CD38, and SARM1) to maintain homeostasis has significant potential in the intervention of age-related diseases and metabolic disorders and offers new research directions for the prevention and treatment of cardiac diseases.

## 6. Summary and Outlook

Heart failure is a complex clinical syndrome, the pathological mechanism of which involves multiple factors, including disturbed energy metabolism, oxidative stress imbalance, activation of the inflammatory response and cell death. In recent years, research has increasingly focused on the central role of mitochondrial dysfunction in the development of heart failure. Mitochondrial dysfunction, as the primary source of energy for cardiomyocytes, results in impaired ATP synthesis and excessive accumulation of ROS, leading to an imbalance in Ca^2+^ homeostasis and dysregulation of mitochondrial dynamics. This, in turn, accelerates the process of cardiomyocyte apoptosis and fibrosis. To address this issue, we propose a treatment for heart failure associated with mitochondrial dysfunction that focuses on increasing NAD^+^ levels as a desirable option. We systematically reviewed the manifestations of mitochondrial dysfunction in heart failure, including mitochondrial kinetic imbalance, oxidative stress injury, dysregulation of mitochondrial autophagy, and disturbance of Ca^2+^ homeostasis. This study further explored the molecular mechanism by which NAD^+^ improves heart failure by modulating mitochondrial function, leading to the proposal of a therapeutic strategy to increase the level of NAD^+^.

**Figure 5:**
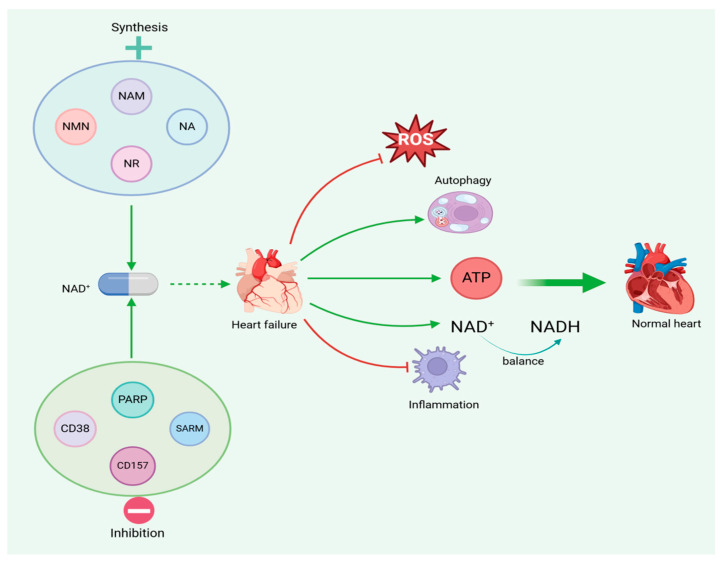
Increased NAD^+^ levels can reduce mitochondrial dysfunction in heart failure, thereby alleviating heart failure.

The levels of NAD^+^, a core molecule involved in energy metabolism and cellular signalling regulation, are significantly reduced in heart failure patients. Research has demonstrated that NAD^+^ can enhance mitochondrial biosynthesis and antioxidant defence, thereby improving mitochondrial function by activating SIRT family deacetylases (e.g., SIRT1 and SIRT3). SIRT1 has been identified as a critical regulator of SGLT2 interactions [233]. SGLT2i have been demonstrated to possess a multitude of cardiovascular and renoprotective effects, thus serving as a novel class of pharmaceutical agents for the treatment of heart failure [234]. Studies have shown that dagliflozin, a representative drug of SGLT2 inhibitors, may enhance cardiomyocyte tolerance under hypoxic conditions by activating the SIRT1 signalling pathway, which in turn leads to an upregulation of both SOD2 and proto-oncogene MYC-mediated cell survival mechanisms [234]. Furthermore, SIRT-1 has been identified as a potential regulator of several epigenetic factors, including those associated with conventional CVD risk. For instance, SIRT1 has been observed to interact with and upregulate AMPKα phosphorylation, which, in turn, activates eNOS and reduces NADPH oxidase activity. This series of events culminates in vasodilation and a reduction in hypertension [235]. In diabetes mellitus, SIRT1 activation is effective in preventing NF-κB, PARP-1, and MMP-9 activation by inhibiting their activation and decreasing the level of histone acetylation in the promoter region of the DNA (cytosine-5)-DNA methyltransferase 1 (DNMT1) promoter to effectively preventing diabetes-induced vasculopathy and mitochondrial damage [236]. In addition, the SIRT1-PGC-1α pathway can negatively regulate the expression of Drp1, inhibit mitochondrial fission, and reduce cardiac dysfunction in diabetic patients [237]. SIRT1 can also inhibit NF-κB signalling through deacetylation, reduce the expression of lectin-like oxLDL receptor-1 (Lox-1), and reduce the uptake of oxLDL, thus preventing the formation of atherosclerosis [238]. SIRT-1 is not only a marker of atherosclerosis. Consequently, SIRT1 can function not only as a potential target of SGLT2i but also as a regulatory factor in conventional CVD risk. In addition, the role of NAD^+^ in regulating the dysregulation of mitochondrial Ca^2+^ homeostasis and reducing cardiomyocyte apoptosis has been demonstrated. Importantly, supplementation with NAD^+^ precursors (e.g., NR, NMN, NAM, and NA) has been shown to significantly improve cardiac function, reduce myocardial fibrosis and delay disease progression in animal models of heart failure. Furthermore, inhibition of the expression of related NAD^+^-degrading enzymes (e.g., PARP, CD38, and SARM) has been shown to increase the NAD^+^ content in cell tissues and prevent the development of the disease in question. Collectively, these studies provide a substantial theoretical foundation for the development of therapeutic interventions targeting NAD^+^ metabolism in the context of heart failure (Figure 5).

## 7. Limitations

Despite noteworthy advancements in the field of NAD^+^ modulation of mitochondrial function to address heart failure, numerous scientific and translational challenges persist, particularly in the evaluation of long-term safety and potential risks associated with NAD^+^ efficacy. Further comprehensive evaluations are imperative to address these challenges. First, clinical trials of NAD^+^ precursors (e.g., NMN, NR) have focused on ageing-related metabolic diseases, and studies on heart failure patients are still at an early stage. Second, extant preclinical studies have chiefly evaluated the therapeutic effects of NMN, NR and NAM on animal models of heart failure. However, experimental animals (e.g., young rodents) generally possess strong endogenous repair potential and immune homeostatic regulation, which is significantly physiologically different from that of the age-dominated human heart failure patient population. This may limit the reliability of the study findings for translation to the clinic. Nevertheless, with a better understanding of the NAD^+^ metabolic network and mitochondrial homeostatic regulatory mechanisms, as well as clinical validation of drug efficacy, intervention strategies targeting NAD^+^ are expected to become important pillars in the comprehensive management of heart failure and open new pathways for improving patient prognosis.

## Figures and Tables

**Table 1:** Core mediators and functions of mitochondrial-mediated heart failure pathology.

Pathomechanism	Core Media	Function	References
Mitochondrial dynamics	MFN1, MFN2, Drp1, OPA1	Regulating mitochondrial fusion (MFN1, MFN2, OPA1) and division (Drp1), the imbalance leads to mitochondrial fragmentation, leading to heart failure	[79]
Mitochondrial autophagy	PINK1, Parkin	PINK1/Parkin is an important component of mitochondrial autophagy mediated by cardiomyocytes. Its deficiency leads to mitochondrial autophagy disorder and promotes cardiomyocyte apoptosis	[97,99,100]
Mitochondrial oxidative stress	SOD, GSH-Px, Catalase	The activity of antioxidant enzymes (SOD scavenging superoxide anion and GPx1/catalase decomposing H_2_O_2_) decreased in heart failure	[112,113,114]
Mitochondrial Ca^2+^ homeostasis	Ca^2+^	Ca^2+^ overload stimulates ROS production, inhibits ATP generation, promotes mitochondrial damage and cardiomyocyte apoptosis, and ultimately leads to heart failure	[125]

**Table 2:** Supplementation of the NAD^+^ precursors to regulate heart failure.

Categories	Dosage	In Vivo/InVitro	Model	Effects	References
NR	400 mg/kg/d	In vivo, In vitro	Mouse HFpEF model	NAD^+^ repletion improved mitochondrial function and reversed HFpEF phenotype	[187]
	500 mg/kg/d	In vivo, In vitro	HFpEF mice	NR repletion fully rescued HFpEF	[170]
	100, 300 or 500 mg/kg	In vivo, In vitro	C57BL/6 mice	NR therapy elevated the NAD^+^ levels, reduced oxidative stress and apoptosis in cardiac tissue, and prevented cardiac injury	[191]
	250 mg, 500 mg, 1000 mg	In vitro	PBMC of stage D HFrEF patients	NR administration enhanced PBMC respiration and reduced proinflammatory cytokine expression in patients with heart failure.	[192]
NMN	500 mg/kg, twice in three days	In vivo, In vitro	Cardiac-restricted complex I-deficient mouse	NMN supplementation increased the NAD^+^/NADH ratio in the cKO hearts, decreased the mitochondrial protein acetylation and improved the sensitivity of the mPTP in the cKO mitochondria	[197]
	500 mg/kg, twice a week	In vivo, In vitro	Friedreich’s ataxia cardiomyopathy mouse model (FXN-KO)	NMN significantly increased cardiac ejection times and improved cardiac energy generation and utilisation in a SIRT3-dependent manner	[198]
	500 mg/kg/d	In vivo, In vitro	Mouse model of contracted TAC	NMN administration improved cardiac mitochondrial function and rescued heart failure	[27]
	500 mg/kg	In vitro	Cardiac of mice undergoing TAC	NMN administration reversed mitochondrial protein hyperacetylation in control mice after TAC and delayed the progression of heart failure	[157]
	A 100× stock in Krebs Henseleit (KH) for 20 min	In vitro	Cardiomyocytes of wild-type C57BL6/J mice and Sirt3^−/−^ mice	NMN stimulated cardiac glycolysis to protect cardiac	[202]
NAM	2.5, 5, 10, or 20 mmol/L	In vitro	Primary rat neonatal cardiomyocytes	NAM pretreatment protected cardiomyocytes by improving mitochondrial stress	[164]
	40 mM	In vivo, In vitro	ZSF1 obese rats	NAM improved diastolic dysfunction in ZSF1 obese rats and alleviated HFpEF risk factors	[207]
	100 μL/tube	In vitro	Primary cardiomyocytes	NAM-induced energy production in hypoxic myocardial cells to protect myocardial cells	[209]
	5, 10, and 20 mM	In vitro	Model of chronic hypoxic myocardial cells	NAM induced autophagy in chronic hypoxic cardiomyocytes and reduced cardiomyocyte apoptosis by regulating the mTOR pathway	[212]
NA	10 mg/kg	In vivo, In vitro	Model of chronic heart failure experimental model	NA significantly reduced the oxidation intensity of cardiomyocyte proteins in heart failure	[218]

Abbreviations: NR: nicotinamide riboside; NMN: β-nicotinamide mononucleotide; NAM: nicotinamide; NA: nicotinic acid; NAD^+^: nicotinamide adenine dinucleotide; NADH: nicotinamide adenine dinucleotide; HFpEF: heart failure with preserved ejection fraction; PBMC: peripheral blood mononuclear cell; TAC: transverse aorta; mPTP: mitochondrial permeability transition pore.

## Data Availability

No data were used for the research described in the article.

## References

[1] Ponikowski P., Voors A.A., Anker S.D., Bueno H., Cleland J.G., Coats A.J., Falk V., González-Juanatey J.R., Harjola V.P., Jankowska E.A. (2016). 2016 ESC Guidelines for the diagnosis and treatment of acute and chronic heart failure: The Task Force for the diagnosis and treatment of acute and chronic heart failure of the European Society of Cardiology (ESC). Developed with the special contribution of the Heart Failure Association (HFA) of the ESC. Eur. J. Heart Fail..

[2] Shimokawa H., Miura M., Nochioka K., Sakata Y. (2015). Heart failure as a general pandemic in Asia. Eur. J. Heart Fail..

[3] He J., Ogden L.G., Bazzano L.A., Vupputuri S., Loria C., Whelton P.K. (2001). Risk factors for congestive heart failure in US men and women: NHANES I epidemiologic follow-up study. Arch. Intern. Med..

[4] Ziaeian B., Fonarow G.C. (2016). Epidemiology and aetiology of heart failure. Nat. Rev. Cardiol..

[5] (2020). Global burden of 369 diseases and injuries in 204 countries and territories, 1990-2019: A systematic analysis for the Global Burden of Disease Study 2019. Lancet.

[6] Savarese G., Becher P.M., Lund L.H., Seferovic P., Rosano G.M.C., Coats A.J.S. (2023). Global burden of heart failure: A comprehensive and updated review of epidemiology. Cardiovasc. Res..

[7] Guha K., McDonagh T. (2013). Heart failure epidemiology: European perspective. Curr. Cardiol. Rev..

[8] Diguet N., Trammell S.A.J., Tannous C., Deloux R., Piquereau J., Mougenot N., Gouge A., Gressette M., Manoury B., Blanc J. (2018). Nicotinamide Riboside Preserves Cardiac Function in a Mouse Model of Dilated Cardiomyopathy. Circulation.

[9] McDonagh T.A., Metra M., Adamo M., Gardner R.S., Baumbach A., Böhm M., Burri H., Butler J., Čelutkienė J., Chioncel O. (2023). 2023 Focused Update of the 2021 ESC Guidelines for the diagnosis and treatment of acute and chronic heart failure. Eur. Heart J..

[10] Lin L., Xu H., Yao Z., Zeng X., Kang L., Li Y., Zhou G., Wang S., Zhang Y., Cheng D. (2024). Jin-Xin-Kang alleviates heart failure by mitigating mitochondrial dysfunction through the Calcineurin/Dynamin-Related Protein 1 signaling pathway. J. Ethnopharmacol..

[11] Popa I.P., Haba MȘ C., Mărănducă M.A., Tănase D.M., Șerban D.N., Șerban L.I., Iliescu R., Tudorancea I. (2022). Modern Approaches for the Treatment of Heart Failure: Recent Advances and Future Perspectives. Pharmaceutics.

[12] Zhong O., Wang J., Tan Y., Lei X., Tang Z. (2022). Effects of NAD^+^ precursor supplementation on glucose and lipid metabolism in humans: A meta-analysis. Nutr. Metab..

[13] Chini C.C.S., Zeidler J.D., Kashyap S., Warner G., Chini E.N. (2021). Evolving concepts in NAD^+^ metabolism. Cell Metab..

[14] Anderson R.M., Bitterman K.J., Wood J.G., Medvedik O., Sinclair D.A. (2003). Nicotinamide and PNC1 govern lifespan extension by calorie restriction in Saccharomyces cerevisiae. Nature.

[15] Xie N., Zhang L., Gao W., Huang C., Huber P.E., Zhou X., Li C., Shen G., Zou B. (2020). NAD^+^ metabolism: Pathophysiologic mechanisms and therapeutic potential. Signal Transduct. Target. Ther..

[16] Fang J., Chen W., Hou P., Liu Z., Zuo M., Liu S., Feng C., Han Y., Li P., Shi Y. (2023). NAD^+^ metabolism-based immunoregulation and therapeutic potential. Cell Biosci..

[17] Hershberger K.A., Martin A.S., Hirschey M.D. (2017). Role of NAD^+^ and mitochondrial sirtuins in cardiac and renal diseases. Nat. Rev. Nephrol..

[18] Li B., Luo C., Chowdhury S., Gao Z.H., Liu J.L. (2013). Parp1 deficient mice are protected from streptozotocin-induced diabetes but not caerulein-induced pancreatitis, independent of the induction of Reg family genes. Regul. Pept..

[19] Mendelsohn A.R., Larrick J.W. (2019). Interacting NAD^+^ and Cell Senescence Pathways Complicate Antiaging Therapies. Rejuvenation Res..

[20] Rotllan N., Camacho M., Tondo M., Diarte-Añazco E.M.G., Canyelles M., Méndez-Lara K.A., Benitez S., Alonso N., Mauricio D., Escolà-Gil J.C. (2021). Therapeutic Potential of Emerging NAD^+^-Increasing Strategies for Cardiovascular Diseases. Antioxidants.

[21] Walker M.A., Tian R. (2018). Raising NAD in Heart Failure: Time to Translate?. Circulation.

[22] Huss J.M., Kelly D.P. (2005). Mitochondrial energy metabolism in heart failure: A question of balance. J. Clin. Investig..

[23] Zhou B., Tian R. (2018). Mitochondrial dysfunction in pathophysiology of heart failure. J. Clin. Investig..

[24] Neubauer S. (2007). The failing heart--an engine out of fuel. N. Engl. J. Med..

[25] Kiyuna L.A., Albuquerque R.P.E., Chen C.H., Mochly-Rosen D., Ferreira J.C.B. (2018). Targeting mitochondrial dysfunction and oxidative stress in heart failure: Challenges and opportunities. Free Radic. Biol. Med..

[26] Wu Y., Pei Z., Qu P. (2024). NAD^+^-A Hub of Energy Metabolism in Heart Failure. Int. J. Med. Sci..

[27] Zhang R., Shen Y., Zhou L., Sangwung P., Fujioka H., Zhang L., Liao X. (2017). Short-term administration of Nicotinamide Mononucleotide preserves cardiac mitochondrial homeostasis and prevents heart failure. J. Mol. Cell. Cardiol..

[28] Matasic D.S., Brenner C., London B. (2018). Emerging potential benefits of modulating NAD^+^ metabolism in cardiovascular disease. Am. J. Physiol. Heart Circ. Physiol..

[29] Arrigo M., Jessup M., Mullens W., Reza N., Shah A.M., Sliwa K., Mebazaa A. (2020). Acute heart failure. Nat. Rev. Dis. Primers.

[30] Cleland J.G.F., Pfeffer M.A., Clark A.L., Januzzi J.L., McMurray J.J.V., Mueller C., Pellicori P., Richards M., Teerlink J.R., Zannad F. (2021). The struggle towards a Universal Definition of Heart Failure-how to proceed?. Eur. Heart J..

[31] Pellicori P., Cleland J.G., Zhang J., Kallvikbacka-Bennett A., Urbinati A., Shah P., Kazmi S., Clark A.L. (2016). Cardiac Dysfunction, Congestion and Loop Diuretics: Their Relationship to Prognosis in Heart Failure. Cardiovasc. Drugs Ther..

[32] Shugg T., Hudmon A., Overholser B.R. (2020). Neurohormonal Regulation of I_Ks_ in Heart Failure: Implications for Ventricular Arrhythmogenesis and Sudden Cardiac Death. J. Am. Heart Assoc..

[33] Lee D.S., Gona P., Vasan R.S., Larson M.G., Benjamin E.J., Wang T.J., Tu J.V., Levy D. (2009). Relation of disease pathogenesis and risk factors to heart failure with preserved or reduced ejection fraction: Insights from the framingham heart study of the national heart, lung, and blood institute. Circulation.

[34] Upadhya B., Kitzman D.W. (2020). Heart failure with preserved ejection fraction: New approaches to diagnosis and management. Clin. Cardiol..

[35] Schwinger R.H.G. (2021). Pathophysiology of heart failure. Cardiovasc. Diagn. Ther..

[36] Golla M.S.G., Hajouli S., Ludhwani D. (2025). Heart Failure and Ejection Fraction. StatPearls.

[37] Kapoor J.R., Kapoor R., Ju C., Heidenreich P.A., Eapen Z.J., Hernandez A.F., Butler J., Yancy C.W., Fonarow G.C. (2016). Precipitating Clinical Factors, Heart Failure Characterization, and Outcomes in Patients Hospitalized With Heart Failure With Reduced, Borderline, and Preserved Ejection Fraction. JACC. Heart Fail..

[38] Tsuji K., Sakata Y., Nochioka K., Miura M., Yamauchi T., Onose T., Abe R., Oikawa T., Kasahara S., Sato M. (2017). Characterization of heart failure patients with mid-range left ventricular ejection fraction-a report from the CHART-2 Study. Eur. J. Heart Fail..

[39] Löfman I., Szummer K., Dahlström U., Jernberg T., Lund L.H. (2017). Associations with and prognostic impact of chronic kidney disease in heart failure with preserved, mid-range, and reduced ejection fraction. Eur. J. Heart Fail..

[40] Wang N., Hales S., Barin E., Tofler G. (2018). Characteristics and outcome for heart failure patients with mid-range ejection fraction. J. Cardiovasc. Med..

[41] Savarese G., Stolfo D., Sinagra G., Lund L.H. (2022). Heart failure with mid-range or mildly reduced ejection fraction. Nat. Rev. Cardiol..

[42] D’Amario D., Migliaro S., Borovac J.A., Restivo A., Vergallo R., Galli M., Leone A.M., Montone R.A., Niccoli G., Aspromonte N. (2019). Microvascular Dysfunction in Heart Failure With Preserved Ejection Fraction. Front. Physiol..

[43] Goidescu C.M., Chiorescu R.M., Diana M.L., Mocan M., Stoia M.A., Anton F.P., Farcaş A.D. (2021). ACE2 and Apelin-13: Biomarkers with a Prognostic Value in Congestive Heart Failure. Dis. Markers.

[44] Chiorescu R.M., Lazar R.D., Buksa S.B., Mocan M., Blendea D. (2022). Biomarkers of Volume Overload and Edema in Heart Failure With Reduced Ejection Fraction. Front. Cardiovasc. Med..

[45] Owan T.E., Hodge D.O., Herges R.M., Jacobsen S.J., Roger V.L., Redfield M.M. (2006). Trends in prevalence and outcome of heart failure with preserved ejection fraction. N. Engl. J. Med..

[46] Satomura H., Wada H., Sakakura K., Kubo N., Ikeda N., Sugawara Y., Ako J., Momomura S. (2012). Congestive heart failure in the elderly: Comparison between reduced ejection fraction and preserved ejection fraction. J. Cardiol..

[47] Tsao C.W., Lyass A., Enserro D., Larson M.G., Ho J.E., Kizer J.R., Gottdiener J.S., Psaty B.M., Vasan R.S. (2018). Temporal Trends in the Incidence of and Mortality Associated With Heart Failure With Preserved and Reduced Ejection Fraction. JACC Heart Fail..

[48] Dassanayaka S., Jones S.P. (2015). Recent Developments in Heart Failure. Circ. Res..

[49] He Y., Huang W., Zhang C., Chen L., Xu R., Li N., Wang F., Han L., Yang M., Zhang D. (2021). Energy metabolism disorders and potential therapeutic drugs in heart failure. Acta Pharm. Sin. B.

[50] Saddik M., Lopaschuk G.D. (1991). Myocardial triglyceride turnover and contribution to energy substrate utilization in isolated working rat hearts. J. Biol. Chem..

[51] Wisneski J.A., Stanley W.C., Neese R.A., Gertz E.W. (1990). Effects of acute hyperglycemia on myocardial glycolytic activity in humans. J. Clin. Investig..

[52] Lee C.F., Tian R. (2015). Mitochondrion as a Target for Heart Failure Therapy- Role of Protein Lysine Acetylation. Circ. J. Off. J. Jpn. Circ. Soc..

[53] Lopaschuk G.D., Ussher J.R., Folmes C.D., Jaswal J.S., Stanley W.C. (2010). Myocardial fatty acid metabolism in health and disease. Physiol. Rev..

[54] Karwi Q.G., Uddin G.M., Ho K.L., Lopaschuk G.D. (2018). Loss of Metabolic Flexibility in the Failing Heart. Front. Cardiovasc. Med..

[55] Brown D.A., Perry J.B., Allen M.E., Sabbah H.N., Stauffer B.L., Shaikh S.R., Cleland J.G., Colucci W.S., Butler J., Voors A.A. (2017). Expert consensus document: Mitochondrial function as a therapeutic target in heart failure. Nat. Rev. Cardiol..

[56] Barth E., Stämmler G., Speiser B., Schaper J. (1992). Ultrastructural quantitation of mitochondria and myofilaments in cardiac muscle from 10 different animal species including man. J. Mol. Cell. Cardiol..

[57] Schaper J., Meiser E., Stämmler G. (1985). Ultrastructural morphometric analysis of myocardium from dogs, rats, hamsters, mice, and from human hearts. Circ. Res..

[58] Butler J., Khan M.S., Anker S.D., Fonarow G.C., Kim R.J., Nodari S., O’Connor C.M., Pieske B., Pieske-Kraigher E., Sabbah H.N. (2020). Effects of Elamipretide on Left Ventricular Function in Patients With Heart Failure With Reduced Ejection Fraction: The PROGRESS-HF Phase 2 Trial. J. Card. Fail..

[59] Casademont J., Miró O. (2002). Electron transport chain defects in heart failure. Heart Fail. Rev..

[60] Quigley A.F., Kapsa R.M., Esmore D., Hale G., Byrne E. (2000). Mitochondrial respiratory chain activity in idiopathic dilated cardiomyopathy. J. Card. Fail..

[61] Jansen J.M. (2017). Rong Tian: Finding What Feeds the Heart. Circ. Res..

[62] Fukushima A., Milner K., Gupta A., Lopaschuk G.D. (2015). Myocardial Energy Substrate Metabolism in Heart Failure: From Pathways to Therapeutic Targets. Curr. Pharm. Des..

[63] Shu H., Peng Y., Hang W., Zhou N., Wang D.W. (2020). Trimetazidine in Heart Failure. Front. Pharmacol..

[64] De Jong K.A., Lopaschuk G.D. (2017). Complex Energy Metabolic Changes in Heart Failure With Preserved Ejection Fraction and Heart Failure With Reduced Ejection Fraction. Can. J. Cardiol..

[65] Fillmore N., Lopaschuk G.D. (2013). Targeting mitochondrial oxidative metabolism as an approach to treat heart failure. Biochim. Biophys. Acta.

[66] Shirakabe A., Zhai P., Ikeda Y., Saito T., Maejima Y., Hsu C.P., Nomura M., Egashira K., Levine B., Sadoshima J. (2016). Drp1-Dependent Mitochondrial Autophagy Plays a Protective Role Against Pressure Overload-Induced Mitochondrial Dysfunction and Heart Failure. Circulation.

[67] Battogtokh G., Choi Y.S., Kang D.S., Park S.J., Shim M.S., Huh K.M., Cho Y.Y., Lee J.Y., Lee H.S., Kang H.C. (2018). Mitochondria-targeting drug conjugates for cytotoxic, anti-oxidizing and sensing purposes: Current strategies and future perspectives. Acta Pharm. Sin. B.

[68] Maack C., Böhm M. (2011). Targeting mitochondrial oxidative stress in heart failure throttling the afterburner. J. Am. Coll. Cardiol..

[69] Wu C., Zhang Z., Zhang W., Liu X. (2022). Mitochondrial dysfunction and mitochondrial therapies in heart failure. Pharmacol. Res..

[70] Tilokani L., Nagashima S., Paupe V., Prudent J. (2018). Mitochondrial dynamics: Overview of molecular mechanisms. Essays Biochem..

[71] Abel E.D. (2018). Mitochondrial dynamics and metabolic regulation in cardiac and skeletal muscle. Trans. Am. Clin. Climatol. Assoc..

[72] Wu D., Dasgupta A., Chen K.H., Neuber-Hess M., Patel J., Hurst T.E., Mewburn J.D., Lima P.D.A., Alizadeh E., Martin A. (2020). Identification of novel dynamin-related protein 1 (Drp1) GTPase inhibitors: Therapeutic potential of Drpitor1 and Drpitor1a in cancer and cardiac ischemia-reperfusion injury. FASEB J. Off. Publ. Fed. Am. Soc. Exp. Biol..

[73] Vona R., Mileo A.M., Matarrese P. (2021). Microtubule-Based Mitochondrial Dynamics as a Valuable Therapeutic Target in Cancer. Cancers.

[74] Meyer J.N., Leuthner T.C., Luz A.L. (2017). Mitochondrial fusion, fission, and mitochondrial toxicity. Toxicology.

[75] Sharma A., Smith H.J., Yao P., Mair W.B. (2019). Causal roles of mitochondrial dynamics in longevity and healthy aging. EMBO Rep..

[76] Quiles J.M., Gustafsson Å.B. (2022). The role of mitochondrial fission in cardiovascular health and disease. Nat. Rev. Cardiol..

[77] Xie J.H., Li Y.Y., Jin J. (2020). The essential functions of mitochondrial dynamics in immune cells. Cell. Mol. Immunol..

[78] Song M., Franco A., Fleischer J.A., Zhang L., Dorn G.W. (2017). Abrogating Mitochondrial Dynamics in Mouse Hearts Accelerates Mitochondrial Senescence. Cell Metab..

[79] Hinton A., Claypool S.M., Neikirk K., Senoo N., Wanjalla C.N., Kirabo A., Williams C.R. (2024). Mitochondrial Structure and Function in Human Heart Failure. Circ. Res..

[80] Sabbah H.N., Gupta R.C., Singh-Gupta V., Zhang K., Lanfear D.E. (2018). Abnormalities of Mitochondrial Dynamics in the Failing Heart: Normalization Following Long-Term Therapy with Elamipretide. Cardiovasc. Drugs Ther..

[81] Varanita T., Soriano M.E., Romanello V., Zaglia T., Quintana-Cabrera R., Semenzato M., Menabò R., Costa V., Civiletto G., Pesce P. (2015). The OPA1-dependent mitochondrial cristae remodeling pathway controls atrophic, apoptotic, and ischemic tissue damage. Cell Metab..

[82] Dorn G.W., Clark C.F., Eschenbacher W.H., Kang M.Y., Engelhard J.T., Warner S.J., Matkovich S.J., Jowdy C.C. (2011). MARF and Opa1 control mitochondrial and cardiac function in Drosophila. Circ. Res..

[83] Wai T., García-Prieto J., Baker M.J., Merkwirth C., Benit P., Rustin P., Rupérez F.J., Barbas C., Ibañez B., Langer T. (2015). Imbalanced OPA1 processing and mitochondrial fragmentation cause heart failure in mice. Science.

[84] Chen L., Liu T., Tran A., Lu X., Tomilov A.A., Davies V., Cortopassi G., Chiamvimonvat N., Bers D.M., Votruba M. (2012). OPA1 mutation and late-onset cardiomyopathy: Mitochondrial dysfunction and mtDNA instability. J. Am. Heart Assoc..

[85] Sun D., Li C., Liu J., Wang Z., Liu Y., Luo C., Chen Y., Wen S. (2019). Expression Profile of microRNAs in Hypertrophic Cardiomyopathy and Effects of microRNA-20 in Inducing Cardiomyocyte Hypertrophy Through Regulating Gene MFN2. DNA Cell Biol..

[86] Ikeda Y., Shirakabe A., Maejima Y., Zhai P., Sciarretta S., Toli J., Nomura M., Mihara K., Egashira K., Ohishi M. (2015). Endogenous Drp1 mediates mitochondrial autophagy and protects the heart against energy stress. Circ. Res..

[87] Vásquez-Trincado C., García-Carvajal I., Pennanen C., Parra V., Hill J.A., Rothermel B.A., Lavandero S. (2016). Mitochondrial dynamics, mitophagy and cardiovascular disease. J. Physiol..

[88] Wu N.N., Zhang Y., Ren J. (2019). Mitophagy, Mitochondrial Dynamics, and Homeostasis in Cardiovascular Aging. Oxidative Med. Cell. Longev..

[89] Ajoolabady A., Aslkhodapasandhokmabad H., Aghanejad A., Zhang Y., Ren J. (2020). Mitophagy Receptors and Mediators: Therapeutic Targets in the Management of Cardiovascular Ageing. Ageing Res. Rev..

[90] Morales P.E., Arias-Durán C., Ávalos-Guajardo Y., Aedo G., Verdejo H.E., Parra V., Lavandero S. (2020). Emerging role of mitophagy in cardiovascular physiology and pathology. Mol. Asp. Med..

[91] Shires S.E., Gustafsson Å.B. (2015). Mitophagy and heart failure. J. Mol. Med..

[92] De Gaetano A., Gibellini L., Zanini G., Nasi M., Cossarizza A., Pinti M. (2021). Mitophagy and Oxidative Stress: The Role of Aging. Antioxidants.

[93] Cadenas E., Davies K.J. (2000). Mitochondrial free radical generation, oxidative stress, and aging. Free Radic. Biol. Med..

[94] Paulus W.J., Tschöpe C. (2013). A novel paradigm for heart failure with preserved ejection fraction: Comorbidities drive myocardial dysfunction and remodeling through coronary microvascular endothelial inflammation. J. Am. Coll. Cardiol..

[95] Kura B., Szeiffova Bacova B., Kalocayova B., Sykora M., Slezak J. (2020). Oxidative Stress-Responsive MicroRNAs in Heart Injury. Int. J. Mol. Sci..

[96] Dolivo D., Weathers P., Dominko T. (2021). Artemisinin and artemisinin derivatives as anti-fibrotic therapeutics. Acta Pharm. Sinica B.

[97] Fan G., Chen M.J., Wei J. (2020). Involvement of phosphatase and tensin homolog-induced putative kinase 1/Parkin-mediated autophagy in angiotensin II-induced cardiac hypertrophy in C57BL/6 mice. J. Int. Med. Res..

[98] Narendra D.P., Jin S.M., Tanaka A., Suen D.F., Gautier C.A., Shen J., Cookson M.R., Youle R.J. (2010). PINK1 is selectively stabilized on impaired mitochondria to activate Parkin. PLoS Biol..

[99] Billia F., Hauck L., Konecny F., Rao V., Shen J., Mak T.W. (2011). PTEN-inducible kinase 1 (PINK1)/Park6 is indispensable for normal heart function. Proc. Natl. Acad. Sci. USA.

[100] Kubli D.A., Zhang X., Lee Y., Hanna R.A., Quinsay M.N., Nguyen C.K., Jimenez R., Petrosyan S., Murphy A.N., Gustafsson A.B. (2013). Parkin protein deficiency exacerbates cardiac injury and reduces survival following myocardial infarction. J. Biol. Chem..

[101] Sies H., Berndt C., Jones D.P. (2017). Oxidative Stress. Annu. Rev. Biochem..

[102] van der Pol A., van Gilst W.H., Voors A.A., van der Meer P. (2019). Treating oxidative stress in heart failure: Past, present and future. Eur. J. Heart Fail..

[103] Liu M., Lv J., Pan Z., Wang D., Zhao L., Guo X. (2022). Mitochondrial dysfunction in heart failure and its therapeutic implications. Front. Cardiovasc. Med..

[104] Bhatti J.S., Bhatti G.K., Reddy P.H. (2017). Mitochondrial dysfunction and oxidative stress in metabolic disorders—A step towards mitochondria based therapeutic strategies. Biochim. Biophys. Acta Mol. Basis Dis..

[105] Zhang M., Perino A., Ghigo A., Hirsch E., Shah A.M. (2013). NADPH oxidases in heart failure: Poachers or gamekeepers?. Antioxid. Redox Signal..

[106] Münzel T., Gori T., Keaney J.F., Maack C., Daiber A. (2015). Pathophysiological role of oxidative stress in systolic and diastolic heart failure and its therapeutic implications. Eur. Heart J..

[107] Li J.M., Shah A.M. (2003). Mechanism of endothelial cell NADPH oxidase activation by angiotensin II. Role of the p47phox subunit. J. Biol. Chem..

[108] Hishikawa K., Lüscher T.F. (1997). Pulsatile stretch stimulates superoxide production in human aortic endothelial cells. Circulation.

[109] Doughan A.K., Harrison D.G., Dikalov S.I. (2008). Molecular mechanisms of angiotensin II-mediated mitochondrial dysfunction: Linking mitochondrial oxidative damage and vascular endothelial dysfunction. Circ. Res..

[110] Cappola T.P., Kass D.A., Nelson G.S., Berger R.D., Rosas G.O., Kobeissi Z.A., Marbán E., Hare J.M. (2001). Allopurinol improves myocardial efficiency in patients with idiopathic dilated cardiomyopathy. Circulation.

[111] Takimoto E., Kass D.A. (2007). Role of oxidative stress in cardiac hypertrophy and remodeling. Hypertension.

[112] Hill M.F., Singal P.K. (1996). Antioxidant and oxidative stress changes during heart failure subsequent to myocardial infarction in rats. Am. J. Pathol..

[113] Khaper N., Singal P.K. (1997). Effects of afterload-reducing drugs on pathogenesis of antioxidant changes and congestive heart failure in rats. J. Am. Coll. Cardiol..

[114] Khaper N., Kaur K., Li T., Farahmand F., Singal P.K. (2003). Antioxidant enzyme gene expression in congestive heart failure following myocardial infarction. Mol. Cell. Biochem..

[115] Rosca M.G., Hoppel C.L. (2013). Mitochondrial dysfunction in heart failure. Heart Fail. Rev..

[116] Al Ghouleh I., Khoo N.K., Knaus U.G., Griendling K.K., Touyz R.M., Thannickal V.J., Barchowsky A., Nauseef W.M., Kelley E.E., Bauer P.M. (2011). Oxidases and peroxidases in cardiovascular and lung disease: New concepts in reactive oxygen species signaling. Free Radic. Biol. Med..

[117] Giorgi C., Danese A., Missiroli S., Patergnani S., Pinton P. (2018). Calcium Dynamics as a Machine for Decoding Signals. Trends Cell Biol..

[118] Stanley W.C., Recchia F.A., Lopaschuk G.D. (2005). Myocardial substrate metabolism in the normal and failing heart. Physiol. Rev..

[119] Lehnart S.E., Maier L.S., Hasenfuss G. (2009). Abnormalities of calcium metabolism and myocardial contractility depression in the failing heart. Heart Fail. Rev..

[120] Burchfield J.S., Xie M., Hill J.A. (2013). Pathological ventricular remodeling: Mechanisms: Part 1 of 2. Circulation.

[121] Weisleder N., Ma J. (2008). Altered Ca^2+^ sparks in aging skeletal and cardiac muscle. Ageing Res. Rev..

[122] Lombardi A.A., Gibb A.A., Arif E., Kolmetzky D.W., Tomar D., Luongo T.S., Jadiya P., Murray E.K., Lorkiewicz P.K., Hajnóczky G. (2019). Mitochondrial calcium exchange links metabolism with the epigenome to control cellular differentiation. Nat. Commun..

[123] Ren J., Pulakat L., Whaley-Connell A., Sowers J.R. (2010). Mitochondrial biogenesis in the metabolic syndrome and cardiovascular disease. J. Mol. Med..

[124] Xu H.X., Cui S.M., Zhang Y.M., Ren J. (2020). Mitochondrial Ca^2+^ regulation in the etiology of heart failure: Physiological and pathophysiological implications. Acta Pharmacol. Sin..

[125] Murphy E., Ardehali H., Balaban R.S., DiLisa F., Dorn G.W., Kitsis R.N., Otsu K., Ping P., Rizzuto R., Sack M.N. (2016). Mitochondrial Function, Biology, and Role in Disease: A Scientific Statement From the American Heart Association. Circ. Res..

[126] Rimessi A., Giorgi C., Pinton P., Rizzuto R. (2008). The versatility of mitochondrial calcium signals: From stimulation of cell metabolism to induction of cell death. Biochim. Biophys. Acta.

[127] Griffiths E.J., Balaska D., Cheng W.H. (2010). The ups and downs of mitochondrial calcium signalling in the heart. Biochim. Biophys. Acta.

[128] La Rovere R.M., Roest G., Bultynck G., Parys J.B. (2016). Intracellular Ca^2+^ signaling and Ca^2+^ microdomains in the control of cell survival, apoptosis and autophagy. Cell Calcium.

[129] East D.A., Campanella M. (2013). Ca^2+^ in quality control: An unresolved riddle critical to autophagy and mitophagy. Autophagy.

[130] Mihaylova M.M., Shaw R.J. (2011). The AMPK signalling pathway coordinates cell growth, autophagy and metabolism. Nat. Cell Biol..

[131] Missiroli S., Bonora M., Patergnani S., Poletti F., Perrone M., Gafà R., Magri E., Raimondi A., Lanza G., Tacchetti C. (2016). PML at Mitochondria-Associated Membranes Is Critical for the Repression of Autophagy and Cancer Development. Cell Rep..

[132] Cárdenas C., Miller R.A., Smith I., Bui T., Molgó J., Müller M., Vais H., Cheung K.H., Yang J., Parker I. (2010). Essential regulation of cell bioenergetics by constitutive InsP3 receptor Ca^2+^ transfer to mitochondria. Cell.

[133] Aryee E.K., Ozkan B., Ndumele C.E. (2023). Heart Failure and Obesity: The Latest Pandemic. Prog. Cardiovasc. Dis..

[134] Nakamura K., Fuster J.J., Walsh K. (2014). Adipokines: A link between obesity and cardiovascular disease. J. Cardiol..

[135] Choi S.E., Fu T., Seok S., Kim D.H., Yu E., Lee K.W., Kang Y., Li X., Kemper B., Kemper J.K. (2013). Elevated microRNA-34a in obesity reduces NAD+ levels and SIRT1 activity by directly targeting NAMPT. Aging Cell.

[136] Rosano G.M., Vitale C., Seferovic P. (2017). Heart Failure in Patients with Diabetes Mellitus. Card. Fail. Rev..

[137] Fukushima A., Kinugawa S., Takada S., Matsushima S., Sobirin M.A., Ono T., Takahashi M., Suga T., Homma T., Masaki Y. (2014). (Pro)renin receptor in skeletal muscle is involved in the development of insulin resistance associated with postinfarct heart failure in mice. Am. J. Physiol. Endocrinol. Metab..

[138] Elendu C., Amaechi D.C., Elendu T.C., Ashna M., Ross-Comptis J., Ansong S.O., Egbunu E.O., Okafor G.C., Jingwa K.A., Akintunde A.A. (2023). Heart failure and diabetes: Understanding the bidirectional relationship. Medicine.

[139] Chiao Y.A., Chakraborty A.D., Light C.M., Tian R., Sadoshima J., Shi X., Gu H., Lee C.F. (2021). NAD^+^ Redox Imbalance in the Heart Exacerbates Diabetic Cardiomyopathy. Circ. Heart Fail..

[140] Hong W., Mo F., Zhang Z., Huang M., Wei X. (2020). Nicotinamide Mononucleotide: A Promising Molecule for Therapy of Diverse Diseases by Targeting NAD+ Metabolism. Front. Cell Dev. Biol..

[141] Li H., Cai Z. (2022). SIRT3 regulates mitochondrial biogenesis in aging-related diseases. J. Biomed. Res..

[142] Li W., Zhou Y., Pang N., Hu Q., Li Q., Sun Y., Ding Y., Gu Y., Xiao Y., Gao M. (2022). NAD Supplement Alleviates Intestinal Barrier Injury Induced by Ethanol Via Protecting Epithelial Mitochondrial Function. Nutrients.

[143] Bugga P., Alam M.J., Kumar R., Pal S., Chattopadyay N., Banerjee S.K. (2022). Sirt3 ameliorates mitochondrial dysfunction and oxidative stress through regulating mitochondrial biogenesis and dynamics in cardiomyoblast. Cell. Signal..

[144] Khan N.A., Auranen M., Paetau I., Pirinen E., Euro L., Forsström S., Pasila L., Velagapudi V., Carroll C.J., Auwerx J. (2014). Effective treatment of mitochondrial myopathy by nicotinamide riboside, a vitamin B3. EMBO Mol. Med..

[145] Wang W., Karamanlidis G., Tian R. (2016). Novel targets for mitochondrial medicine. Sci. Transl. Med..

[146] Imai S., Guarente L. (2014). NAD+ and sirtuins in aging and disease. Trends Cell Biol..

[147] Hamaidi I., Kim S. (2022). Sirtuins are crucial regulators of T cell metabolism and functions. Exp. Mol. Med..

[148] Vaquero A., Scher M., Lee D., Erdjument-Bromage H., Tempst P., Reinberg D. (2004). Human SirT1 interacts with histone H1 and promotes formation of facultative heterochromatin. Mol. Cell.

[149] Das A., Huang G.X., Bonkowski M.S., Longchamp A., Li C., Schultz M.B., Kim L.J., Osborne B., Joshi S., Lu Y. (2018). Impairment of an Endothelial NAD^+^-H_2_S Signaling Network Is a Reversible Cause of Vascular Aging. Cell.

[150] Cantó C., Gerhart-Hines Z., Feige J.N., Lagouge M., Noriega L., Milne J.C., Elliott P.J., Puigserver P., Auwerx J. (2009). AMPK regulates energy expenditure by modulating NAD^+^ metabolism and SIRT1 activity. Nature.

[151] Berk B.C., Fujiwara K., Lehoux S. (2007). ECM remodeling in hypertensive heart disease. J. Clin. Investig..

[152] Hill J.A., Olson E.N. (2008). Cardiac plasticity. N. Engl. J. Med..

[153] Nakamura M., Odanovic N., Nakada Y., Dohi S., Zhai P., Ivessa A., Yang Z., Abdellatif M., Sadoshima J. (2021). Dietary carbohydrates restriction inhibits the development of cardiac hypertrophy and heart failure. Cardiovasc. Res..

[154] Lombard D.B., Alt F.W., Cheng H.L., Bunkenborg J., Streeper R.S., Mostoslavsky R., Kim J., Yancopoulos G., Valenzuela D., Murphy A. (2007). Mammalian Sir2 homolog SIRT3 regulates global mitochondrial lysine acetylation. Mol. Cell. Biol..

[155] Finley L.W., Haas W., Desquiret-Dumas V., Wallace D.C., Procaccio V., Gygi S.P., Haigis M.C. (2011). Succinate dehydrogenase is a direct target of sirtuin 3 deacetylase activity. PLoS ONE.

[156] Pillai V.B., Sundaresan N.R., Kim G., Gupta M., Rajamohan S.B., Pillai J.B., Samant S., Ravindra P.V., Isbatan A., Gupta M.P. (2010). Exogenous NAD blocks cardiac hypertrophic response via activation of the SIRT3-LKB1-AMP-activated kinase pathway. J. Biol. Chem..

[157] Lee C.F., Chavez J.D., Garcia-Menendez L., Choi Y., Roe N.D., Chiao Y.A., Edgar J.S., Goo Y.A., Goodlett D.R., Bruce J.E. (2016). Normalization of NAD+ Redox Balance as a Therapy for Heart Failure. Circulation.

[158] Noh M.R., Kong M.J., Han S.J., Kim J.I., Park K.M. (2020). Isocitrate dehydrogenase 2 deficiency aggravates prolonged high-fat diet intake-induced hypertension. Redox Biol..

[159] Kim H., Lee Y.D., Kim H.J., Lee Z.H., Kim H.H. (2017). SOD2 and Sirt3 Control Osteoclastogenesis by Regulating Mitochondrial ROS. J. Bone Miner. Res. Off. J. Am. Soc. Bone Miner. Res..

[160] Yu W., Dittenhafer-Reed K.E., Denu J.M. (2012). SIRT3 protein deacetylates isocitrate dehydrogenase 2 (IDH2) and regulates mitochondrial redox status. J. Biol. Chem..

[161] Hafner A.V., Dai J., Gomes A.P., Xiao C.Y., Palmeira C.M., Rosenzweig A., Sinclair D.A. (2010). Regulation of the mPTP by SIRT3-mediated deacetylation of CypD at lysine 166 suppresses age-related cardiac hypertrophy. Aging.

[162] Rimessi A., Bonora M., Marchi S., Patergnani S., Marobbio C.M., Lasorsa F.M., Pinton P. (2013). Perturbed mitochondrial Ca^2+^ signals as causes or consequences of mitophagy induction. Autophagy.

[163] Liu Y., Landick R., Raman S. (2019). A Regulatory NADH/NAD+ Redox Biosensor for Bacteria. ACS Synth. Biol..

[164] Tong D.L., Zhang D.X., Xiang F., Teng M., Jiang X.P., Hou J.M., Zhang Q., Huang Y.S. (2012). Nicotinamide pretreatment protects cardiomyocytes against hypoxia-induced cell death by improving mitochondrial stress. Pharmacology.

[165] Nakamura M., Bhatnagar A., Sadoshima J. (2012). Overview of pyridine nucleotides review series. Circ. Res..

[166] de Picciotto N.E., Gano L.B., Johnson L.C., Martens C.R., Sindler A.L., Mills K.F., Imai S., Seals D.R. (2016). Nicotinamide mononucleotide supplementation reverses vascular dysfunction and oxidative stress with aging in mice. Aging Cell.

[167] Klimova N., Long A., Kristian T. (2019). Nicotinamide mononucleotide alters mitochondrial dynamics by SIRT3-dependent mechanism in male mice. J. Neurosci. Res..

[168] Mehmel M., Jovanović N., Spitz U. (2020). Nicotinamide Riboside-The Current State of Research and Therapeutic Uses. Nutrients.

[169] Bieganowski P., Brenner C. (2004). Discoveries of nicotinamide riboside as a nutrient and conserved NRK genes establish a Preiss-Handler independent route to NAD+ in fungi and humans. Cell.

[170] Koay Y.C., Liu R.P., McIntosh B., Vigder N., Lauren S., Bai A.Y., Tomita S., Li D., Harney D., Hunter B. (2024). The Efficacy of Risk Factor Modification Compared to NAD^+^ Repletion in Diastolic Heart Failure. JACC Basic Transl. Sci..

[171] Hu L., Guo Y., Song L., Wen H., Sun N., Wang Y., Qi B., Liang Q., Geng J., Liu X. (2022). Nicotinamide riboside promotes Mfn2-mediated mitochondrial fusion in diabetic hearts through the SIRT1-PGC1α-PPARα pathway. Free Radic. Biol. Med..

[172] Abdellatif M., Vasques-Nóvoa F., Trummer-Herbst V., Durand S., Koser F., Islam M., Nah J., Sung E.A., Feng R., Aprahamian F. (2025). Autophagy is required for the therapeutic effects of the NAD+ precursor nicotinamide in obesity-related heart failure with preserved ejection fraction. Eur. Heart J..

[173] Broeks M.H., Meijer N.W.F., Westland D., Bosma M., Gerrits J., German H.M., Ciapaite J., van Karnebeek C.D.M., Wanders R.J.A., Zwartkruis F.J.T. (2023). The malate-aspartate shuttle is important for de novo serine biosynthesis. Cell Rep..

[174] Zuurbier C.J., Bertrand L., Beauloye C.R., Andreadou I., Ruiz-Meana M., Jespersen N.R., Kula-Alwar D., Prag H.A., Eric Botker H., Dambrova M. (2020). Cardiac metabolism as a driver and therapeutic target of myocardial infarction. J. Cell. Mol. Med..

[175] Nielsen T.T., Støttrup N.B., Løfgren B., Bøtker H.E. (2011). Metabolic fingerprint of ischaemic cardioprotection: Importance of the malate-aspartate shuttle. Cardiovasc. Res..

[176] Sun W., Liu Q., Leng J., Zheng Y., Li J. (2015). The role of Pyruvate Dehydrogenase Complex in cardiovascular diseases. Life Sci..

[177] Ussher J.R., Jaswal J.S., Lopaschuk G.D. (2012). Pyridine nucleotide regulation of cardiac intermediary metabolism. Circ. Res..

[178] Wang L., Quan N., Sun W., Chen X., Cates C., Rousselle T., Zhou X., Zhao X., Li J. (2018). Cardiomyocyte-specific deletion of Sirt1 gene sensitizes myocardium to ischaemia and reperfusion injury. Cardiovasc. Res..

[179] Riess M.L., Camara A.K., Chen Q., Novalija E., Rhodes S.S., Stowe D.F. (2002). Altered NADH and improved function by anesthetic and ischemic preconditioning in guinea pig intact hearts. Am. J. Physiol. Heart Circ. Physiol..

[180] Sims C.A., Guan Y., Mukherjee S., Singh K., Botolin P., Davila A., Baur J.A. (2018). Nicotinamide mononucleotide preserves mitochondrial function and increases survival in hemorrhagic shock. JCI Insight.

[181] Lee C.F., Caudal A., Abell L., Nagana Gowda G.A., Tian R. (2019). Targeting NAD^+^ Metabolism as Interventions for Mitochondrial Disease. Sci. Rep..

[182] Rajman L., Chwalek K., Sinclair D.A. (2018). Therapeutic Potential of NAD-Boosting Molecules: The In Vivo Evidence. Cell Metab..

[183] Airhart S.E., Shireman L.M., Risler L.J., Anderson G.D., Nagana Gowda G.A., Raftery D., Tian R., Shen D.D., O’Brien K.D. (2017). An open-label, non-randomized study of the pharmacokinetics of the nutritional supplement nicotinamide riboside (NR) and its effects on blood NAD+ levels in healthy volunteers. PLoS ONE.

[184] Trammell S.A., Schmidt M.S., Weidemann B.J., Redpath P., Jaksch F., Dellinger R.W., Li Z., Abel E.D., Migaud M.E., Brenner C. (2016). Nicotinamide riboside is uniquely and orally bioavailable in mice and humans. Nat. Commun..

[185] Smyrnias I., Gray S.P., Okonko D.O., Sawyer G., Zoccarato A., Catibog N., López B., González A., Ravassa S., Díez J. (2019). Cardioprotective Effect of the Mitochondrial Unfolded Protein Response During Chronic Pressure Overload. J. Am. Coll. Cardiol..

[186] Liu X., Zhang Y., Deng Y., Yang L., Ou W., Xie M., Ding L., Jiang C., Yu H., Li Q. (2022). Mitochondrial protein hyperacetylation underpins heart failure with preserved ejection fraction in mice. J. Mol. Cell. Cardiol..

[187] Tong D., Schiattarella G.G., Jiang N., Altamirano F., Szweda P.A., Elnwasany A., Lee D.I., Yoo H., Kass D.A., Szweda L.I. (2021). NAD^+^ Repletion Reverses Heart Failure With Preserved Ejection Fraction. Circ. Res..

[188] Oka T., Hikoso S., Yamaguchi O., Taneike M., Takeda T., Tamai T., Oyabu J., Murakawa T., Nakayama H., Nishida K. (2012). Mitochondrial DNA that escapes from autophagy causes inflammation and heart failure. Nature.

[189] Zang Q.S., Sadek H., Maass D.L., Martinez B., Ma L., Kilgore J.A., Williams N.S., Frantz D.E., Wigginton J.G., Nwariaku F.E. (2012). Specific inhibition of mitochondrial oxidative stress suppresses inflammation and improves cardiac function in a rat pneumonia-related sepsis model. Am. J. Physiol. Heart Circ. Physiol..

[190] Dan Dunn J., Alvarez L.A., Zhang X., Soldati T. (2015). Reactive oxygen species and mitochondria: A nexus of cellular homeostasis. Redox Biol..

[191] Hong G., Zheng D., Zhang L., Ni R., Wang G., Fan G.C., Lu Z., Peng T. (2018). Administration of nicotinamide riboside prevents oxidative stress and organ injury in sepsis. Free Radic. Biol. Med..

[192] Zhou B., Wang D.D., Qiu Y., Airhart S., Liu Y., Stempien-Otero A., O’Brien K.D., Tian R. (2020). Boosting NAD level suppresses inflammatory activation of PBMCs in heart failure. J. Clin. Investig..

[193] Nakagawa T., Guarente L. (2014). SnapShot: Sirtuins, NAD, and aging. Cell Metab..

[194] Mills K.F., Yoshida S., Stein L.R., Grozio A., Kubota S., Sasaki Y., Redpath P., Migaud M.E., Apte R.S., Uchida K. (2016). Long-Term Administration of Nicotinamide Mononucleotide Mitigates Age-Associated Physiological Decline in Mice. Cell Metab..

[195] Yoshino J., Baur J.A., Imai S.I. (2018). NAD^+^ Intermediates: The Biology and Therapeutic Potential of NMN and NR. Cell Metab..

[196] Yagi M., Do Y., Hirai H., Miki K., Toshima T., Fukahori Y., Setoyama D., Abe C., Nabeshima Y.I., Kang D. (2023). Improving lysosomal ferroptosis with NMN administration protects against heart failure. Life Sci. Alliance.

[197] Karamanlidis G., Lee C.F., Garcia-Menendez L., Kolwicz S.C., Suthammarak W., Gong G., Sedensky M.M., Morgan P.G., Wang W., Tian R. (2013). Mitochondrial complex I deficiency increases protein acetylation and accelerates heart failure. Cell Metab..

[198] Martin A.S., Abraham D.M., Hershberger K.A., Bhatt D.P., Mao L., Cui H., Liu J., Liu X., Muehlbauer M.J., Grimsrud P.A. (2017). Nicotinamide mononucleotide requires SIRT3 to improve cardiac function and bioenergetics in a Friedreich’s ataxia cardiomyopathy model. JCI Insight.

[199] Zhang Y., Bharathi S.S., Beck M.E., Goetzman E.S. (2019). The fatty acid oxidation enzyme long-chain acyl-CoA dehydrogenase can be a source of mitochondrial hydrogen peroxide. Redox Biol..

[200] Nascimben L., Ingwall J.S., Lorell B.H., Pinz I., Schultz V., Tornheim K., Tian R. (2004). Mechanisms for increased glycolysis in the hypertrophied rat heart. Hypertension.

[201] Tran D.H., Wang Z.V. (2019). Glucose Metabolism in Cardiac Hypertrophy and Heart Failure. J. Am. Heart Assoc..

[202] Nadtochiy S.M., Wang Y.T., Nehrke K., Munger J., Brookes P.S. (2018). Cardioprotection by nicotinamide mononucleotide (NMN): Involvement of glycolysis and acidic pH. J. Mol. Cell. Cardiol..

[203] Jaconello P. (1992). Niacin versus niacinamide. CMAJ Can. Med. Assoc. J. J. L’association Medicale Can..

[204] Jung M., Lee K.M., Im Y., Seok S.H., Chung H., Kim D.Y., Han D., Lee C.H., Hwang E.H., Park S.Y. (2022). Nicotinamide (niacin) supplement increases lipid metabolism and ROS-induced energy disruption in triple-negative breast cancer: Potential for drug repositioning as an anti-tumor agent. Mol. Oncol..

[205] Covarrubias A.J., Perrone R., Grozio A., Verdin E. (2021). NAD^+^ metabolism and its roles in cellular processes during ageing. Nat. Rev. Mol. Cell Biol..

[206] van Ommen A., Canto E.D., Cramer M.J., Rutten F.H., Onland-Moret N.C., Ruijter H.M.D. (2022). Diastolic dysfunction and sex-specific progression to HFpEF: Current gaps in knowledge and future directions. BMC Med..

[207] Abdellatif M., Trummer-Herbst V., Koser F., Durand S., Adão R., Vasques-Nóvoa F., Freundt J.K., Voglhuber J., Pricolo M.R., Kasa M. (2021). Nicotinamide for the treatment of heart failure with preserved ejection fraction. Sci. Transl. Med..

[208] Sharma A., Anand S.K., Singh N., Dwivedi U.N., Kakkar P. (2023). AMP-activated protein kinase: An energy sensor and survival mechanism in the reinstatement of metabolic homeostasis. Exp. Cell Res..

[209] Lai Y.F., Wang L., Liu W.Y. (2019). Nicotinamide pretreatment alleviates mitochondrial stress and protects hypoxic myocardial cells via AMPK pathway. Eur. Rev. Med. Pharmacol. Sci..

[210] Kim Y.C., Guan K.L. (2015). mTOR: A pharmacologic target for autophagy regulation. J. Clin. Investig..

[211] Munson M.J., Ganley I.G. (2015). MTOR, PIK3C3, and autophagy: Signaling the beginning from the end. Autophagy.

[212] Li W., Zhu L., Ruan Z.B., Wang M.X., Ren Y., Lu W. (2019). Nicotinamide protects chronic hypoxic myocardial cells through regulating mTOR pathway and inducing autophagy. Eur. Rev. Med. Pharmacol. Sci..

[213] Yang R., Zhu M., Fan S., Zhang J. (2024). Niacin intake and mortality (total and cardiovascular disease) in patients with cardiovascular disease: Insights from NHANES 2003–2018. Nutr. J..

[214] Gasperi V., Sibilano M., Savini I., Catani M.V. (2019). Niacin in the Central Nervous System: An Update of Biological Aspects and Clinical Applications. Int. J. Mol. Sci..

[215] Saeedi R., Frohlich J. (2016). Lipoprotein (a), an independent cardiovascular risk marker. Clin. Diabetes Endocrinol..

[216] Lavigne P.M., Karas R.H. (2013). The current state of niacin in cardiovascular disease prevention: A systematic review and meta-regression. J. Am. Coll. Cardiol..

[217] Fu Y., Xu C., Wu G. (2024). Dietary niacin Intake and its association with all-cause and cardiovascular mortality rates in individuals with metabolic syndrome. Nutr. J..

[218] Nizhenkovska I., Narokha V., Kuznetsova O. (2018). Effects of nicotinic acid on protein oxidative modifications in experimental chronic heart failure. Farmacia.

[219] Chini E.N., Chini C.C.S., Espindola Netto J.M., de Oliveira G.C., van Schooten W. (2018). The Pharmacology of CD38/NADase: An Emerging Target in Cancer and Diseases of Aging. Trends Pharmacol. Sci..

[220] Guan X.H., Hong X., Zhao N., Liu X.H., Xiao Y.F., Chen T.T., Deng L.B., Wang X.L., Wang J.B., Ji G.J. (2017). CD38 promotes angiotensin II-induced cardiac hypertrophy. J. Cell. Mol. Med..

[221] Boslett J., Hemann C., Zhao Y.J., Lee H.C., Zweier J.L. (2017). Luteolinidin Protects the Postischemic Heart through CD38 Inhibition with Preservation of NAD(P)(H). J. Pharmacol. Exp. Ther..

[222] Kellenberger E., Kuhn I., Schuber F., Muller-Steffner H. (2011). Flavonoids as inhibitors of human CD38. Bioorganic Med. Chem. Lett..

[223] Espírito-Santo D.A., Cordeiro G.S., Santos L.S., Silva R.T., Pereira M.U., Matos R.J.B., Boaventura G.T., Barreto-Medeiros J.M. (2023). Cardioprotective effect of the quercetin on cardiovascular remodeling and atherosclerosis in rodents fed a high-fat diet: A systematic review. Chem.-Biol. Interact..

[224] Camacho-Pereira J., Tarragó M.G., Chini C.C.S., Nin V., Escande C., Warner G.M., Puranik A.S., Schoon R.A., Reid J.M., Galina A. (2016). CD38 Dictates Age-Related NAD Decline and Mitochondrial Dysfunction through an SIRT3-Dependent Mechanism. Cell Metab..

[225] Escande C., Nin V., Price N.L., Capellini V., Gomes A.P., Barbosa M.T., O’Neil L., White T.A., Sinclair D.A., Chini E.N. (2013). Flavonoid apigenin is an inhibitor of the NAD^+^ ase CD38: Implications for cellular NAD^+^ metabolism, protein acetylation, and treatment of metabolic syndrome. Diabetes.

[226] Ogura Y., Kitada M., Xu J., Monno I., Koya D. (2020). CD38 inhibition by apigenin ameliorates mitochondrial oxidative stress through restoration of the intracellular NAD^+^/NADH ratio and Sirt3 activity in renal tubular cells in diabetic rats. Aging.

[227] Tarragó M.G., Chini C.C.S., Kanamori K.S., Warner G.M., Caride A., de Oliveira G.C., Rud M., Samani A., Hein K.Z., Huang R. (2018). A Potent and Specific CD38 Inhibitor Ameliorates Age-Related Metabolic Dysfunction by Reversing Tissue NAD^+^ Decline. Cell Metab..

[228] Boslett J., Reddy N., Alzarie Y.A., Zweier J.L. (2019). Inhibition of CD38 with the Thiazoloquin(az)olin(on)e 78c Protects the Heart against Postischemic Injury. J. Pharmacol. Exp. Ther..

[229] Summers D.W., Gibson D.A., DiAntonio A., Milbrandt J. (2016). SARM1-specific motifs in the TIR domain enable NAD+ loss and regulate injury-induced SARM1 activation. Proc. Natl. Acad. Sci. USA.

[230] Gerdts J., Brace E.J., Sasaki Y., DiAntonio A., Milbrandt J. (2015). SARM1 activation triggers axon degeneration locally via NAD^+^ destruction. Science.

[231] Huang S.M., Mishina Y.M., Liu S., Cheung A., Stegmeier F., Michaud G.A., Charlat O., Wiellette E., Zhang Y., Wiessner S. (2009). Tankyrase inhibition stabilizes axin and antagonizes Wnt signalling. Nature.

[232] Tentori L., Ricci-Vitiani L., Muzi A., Ciccarone F., Pelacchi F., Calabrese R., Runci D., Pallini R., Caiafa P., Graziani G. (2014). Pharmacological inhibition of poly(ADP-ribose) polymerase-1 modulates resistance of human glioblastoma stem cells to temozolomide. BMC Cancer.

[233] Wicik Z., Nowak A., Jarosz-Popek J., Wolska M., Eyileten C., Siller-Matula J.M., von Lewinski D., Sourij H., Filipiak K.J., Postuła M. (2022). Characterization of the SGLT2 Interaction Network and Its Regulation by SGLT2 Inhibitors: A Bioinformatic Analysis. Front. Pharmacol..

[234] Lin Y.H., Tsai W.C., Chiu C.C., Chi N.Y., Liu Y.H., Huang T.C., Wu W.T., Lin T.H., Lai W.T., Sheu S.H. (2024). The Beneficial Effect of the SGLT2 Inhibitor Dapagliflozin in Alleviating Acute Myocardial Infarction-Induced Cardiomyocyte Injury by Increasing the Sirtuin Family SIRT1/SIRT3 and Cascade Signaling. Int. J. Mol. Sci..

[235] Gao D., Zuo Z., Tian J., Ali Q., Lin Y., Lei H., Sun Z. (2016). Activation of SIRT1 Attenuates Klotho Deficiency-Induced Arterial Stiffness and Hypertension by Enhancing AMP-Activated Protein Kinase Activity. Hypertension.

[236] Hammer S.S., Vieira C.P., McFarland D., Sandler M., Levitsky Y., Dorweiler T.F., Lydic T.A., Asare-Bediako B., Adu-Agyeiwaah Y., Sielski M.S. (2021). Fasting and fasting-mimicking treatment activate SIRT1/LXRα and alleviate diabetes-induced systemic and microvascular dysfunction. Diabetologia.

[237] Ding M., Feng N., Tang D., Feng J., Li Z., Jia M., Liu Z., Gu X., Wang Y., Fu F. (2018). Melatonin prevents Drp1-mediated mitochondrial fission in diabetic hearts through SIRT1-PGC1α pathway. J. Pineal Res..

[238] Stein S., Lohmann C., Schäfer N., Hofmann J., Rohrer L., Besler C., Rothgiesser K.M., Becher B., Hottiger M.O., Borén J. (2010). SIRT1 decreases Lox-1-mediated foam cell formation in atherogenesis. Eur. Heart J..

